# Imaging tumor acidosis: a survey of the available techniques for mapping *in vivo* tumor pH

**DOI:** 10.1007/s10555-019-09782-9

**Published:** 2019-02-14

**Authors:** Annasofia Anemone, Lorena Consolino, Francesca Arena, Martina Capozza, Dario Livio Longo

**Affiliations:** 1 Molecular Imaging Center, Department of Molecular Biotechnology and Health Sciences, University of Turin, Via Nizza 52, Turin, Italy; 2 Institute of Biostructures and Bioimaging (IBB), Italian National Research Council (CNR), Via Nizza 52, Turin, Italy; 3 Center for Preclinical Imaging, Department of Molecular Biotechnology and Health Sciences, University of Turin, Via Ribes 5, Colleretto Giacosa, Italy

**Keywords:** pH imaging, Tumor acidosis, Magnetic resonance imaging, Chemical Exchange Saturation Transfer (CEST) imaging, Iopamidol, pH-responsive probes

## Abstract

Cancer cells are characterized by a metabolic shift in cellular energy production, orchestrated by the transcription factor HIF-1α, from mitochondrial oxidative phosphorylation to increased glycolysis, regardless of oxygen availability (Warburg effect). The constitutive upregulation of glycolysis leads to an overproduction of acidic metabolic products, resulting in enhanced acidification of the extracellular pH (pHe ~ 6.5), which is a salient feature of the tumor microenvironment. Despite the importance of pH and tumor acidosis, there is currently no established clinical tool available to image the spatial distribution of tumor pHe. The purpose of this review is to describe various imaging modalities for measuring intracellular and extracellular tumor pH. For each technique, we will discuss main advantages and limitations, pH accuracy and sensitivity of the applied pH-responsive probes and potential translatability to the clinic. Particular attention is devoted to methods that can provide pH measurements at high spatial resolution useful to address the task of tumor heterogeneity and to studies that explored tumor pH imaging for assessing treatment response to anticancer therapies.

## Introduction

Solid tumors are characterized by a highly heterogeneous microenvironment, resulting from the combination of poor vascular perfusion and regional hypoxia [1]. Metabolic adaptation represents a canonical response of tumor cells to survive, orchestrated by the transcription factor HIF-1α, which modulates genes involved in angiogenesis, glycolysis, proliferation, and metastasis. This metabolic shift is characterized by elevated glycolysis and lactate production, regardless of oxygen availability (Warburg effect) [2, 3]. The constitutive upregulation of glycolysis leads to the exaggerated generation of metabolites, including acidic products such as lactate and protons that, upon accumulation in the cytoplasm, might result in intracellular acidosis. Therefore, tumor cells require additional activities in order to maintain an intracellular pH (pHi) compatible with the biochemical processes typical of cells characterized by high proliferation rates [4, 5]. This task is taken up by several and redundant families of proton transporters that excrete lactate molecules and protons into the extracellular-extravascular compartment, which induces extracellular acidification, resulting in the reversed extracellular pH (pHe) gradient in tumors in comparison to healthy tissues [6].

Clinical investigations support the view that the acidic microenvironment in tumors results in less favorable prognosis associated with increased metastatic potential and resistance to chemo- and radiotherapy [7]. Therefore, acid production (or extracellular acidification) is even more important than the altered glucose metabolism [8, 9]. Because survival in the tumor microenvironment depends on the control of pH, interference with pH regulating systems is considered a relevant therapeutic goal [10]. Thus, extracellular acidosis of solid tumors acquires a prognostic value and therapeutic manipulation of tumor acidosis might represent a novel strategy for a successful treatment of cancer [11, 12].

On the basis of the above considerations, the need for advanced medical imaging approaches, based on mapping of tumor pHe that allows early detection of treatment effect upon the impairment of tumor pH dynamics, is urgently needed. The development/exploitation of imaging-based procedures able to quantitatively measure tumor pHe may provide noninvasive additional information to currently used nuclear medicine techniques (^18^F-FDG/PET) for cancer assessment. If pH regulation is an essential component of tumor cell survival and its impairment may halt proliferation of the primary tumor and formation of metastases, a tool able to noninvasively quantify tumor pHe appears to have optimal chances to be translated to the clinic.

This article surveys the imaging-related methods that have been proposed, and validated *in vivo*, for measuring tumor acidosis, including magnetic resonance imaging (MRI) and spectroscopy (MRS), nuclear medicine (positron emission tomography, PET), electron paramagnetic resonance (EPR), optical imaging (OI), and photoacoustic imaging (PAI). For each method, the basic principles are described together with the most representative pH-sensitive probes and approaches that have been exploited for attaining *in vivo* tumor pH maps.

## Magnetic resonance imaging methods

### Magnetic resonance spectroscopy

Magnetic resonance spectroscopy (MRS) has been proposed in the early days of *in vivo* nuclear magnetic resonance (NMR) applications as a direct approach to the detection and quantification of metabolites in living tissues. In the oncological field, MRS allows the assessment of abnormal metabolic profiles that may act as useful prognostic biomarkers. In addition to metabolites, MRS has also been exploited for assessing pHi and pHe compartments of tumors cells by combining acceptable sensitivity threshold with spatial resolution. Intense efforts have been devoted to design suitable pH reporters with the aim of satisfying the criteria of favorable pharmacokinetics, p*K*a suitable for the physiological pH range, good sensitivity, and low toxicity. The measurement of pH by MRS is based on the chemical shift difference between a pH-dependent resonance and a pH-independent peak taken as reference. ^31^P, ^1^H, and ^19^F have been the most investigated NMR active nuclei for selecting pH-dependent resonances containing species.

#### MRS—^31^P

^31^P MRS has been the first technique applied for measuring pH in tumors. Phosphorus-31 is 100% abundant and several phosphorus-based metabolites are intracellularly present at a concentration of 0.1–5.0 mM. Among them, inorganic phosphate (Pi) is routinely used for measuring pH *in vivo* due to the dependence of its chemical shift on pH changes in the physiological range (p*K*a ~ 6.8). As pH-independent reference peaks, the signals from endogenous nucleoside triphosphates (NTP) and phosphocreatine (PCr) are usually considered. Concerns regarding the use of different reference spectra on pH determination have been raised; however, a recent comparative study involving healthy volunteers and patients with non-Hodgkin’s lymphoma demonstrated that the direct Pi-αATP method is more reliable for measuring pH in tumors, showing low variation among patients and reasonable repeatability [13]. Considering that intracellular Pi is present at a concentration of 2–3 mM, whereas in the extracellular compartment is present at a concentration of 1 mM; it can be calculated that, for an extracellular volume below 55%, most of the Pi-MRS-based signal is coming from the intracellular compartment, thus indicating that the observed shift of Pi is essentially a reporter of intracellular pH [14]. This statement has been validated *in vivo* by exploiting the intracellular pH reporter 2-deoxyglucose (2DG), that is phosphorylated to 2DG6P and accumulates within cells overcoming the glycolytic process [15]. ^31^P-MRS of fibrosarcoma xenograft tumors revealed a good correspondence between pH values obtained from Pi and from 2DG6P measurements, confirming that Pi-MRS measurements definitely report on intracellular pH. To supply the lack of extracellular pH reporter probes for ^31^P-MRS, exogenous phosphonate agents have also been developed. Despite the fact that several extracellular phosphonate-based probes showed good characteristics [16], most *in vivo* applications have historically involved the use of 3-aminopropylphosphonate (3-APP). This compound shows a p*K*a in the physiological range and accurately reports the pHe with little influence of temperature and ion effect [17]. Therefore, simultaneous acquisition of Pi and 3-APP can be combined for assessing the pH values within tissue compartments and quantitative parameters can be extrapolated for an extensive characterization [18]. ^31^P-MRS measurements elucidated *in vivo* the concept of the cellular pH gradient of tumors, indicating that intracellular pH in tumor is usually more alkaline in comparison to normal tissue, whereas extracellular pH is generally more acidic. This peculiar information has been exploited in several studies aiming at reverting the acidic-base pH gradient as a potential approach for treating cancer. This idea is based on the fact that the kinetic uptake of drugs strongly depends on their ionization state in relation to a specific pHi/pHe condition [19]. Several *in vivo* investigations showed increased cytotoxic activity of chemotherapeutic drugs as mitoxantrone and doxorubicin upon induced tumor alkalinization with sodium bicarbonate, which raises the extracellular pH of 0.4–0.8 units. [20, 21] Moreover, inhibitors of mitochondrial metabolism in combination with hyperglycemic conditions induced selective acidification of human melanoma xenografts, with a significant decrease of both intra and extracellular pH [22]. Furthermore, ^31^P-MRS approach was recently used in a mouse model to evaluate early intracellular pH changes upon antiangiogenic treatment of recurrent glioblastoma [23]. This approach can therefore provide assessments of both intra- and extracellular tumor pH by combining endogenous and exogenous 31P-containing molecules. However, the potential neurotoxicity of 3-APP (analog of the γ-aminobutyric acid neurotransmitter) in the presence of compromised blood brain barriers is a concern for human use and the low spatial resolution and long acquisition times combined with the requirement of dedicated coils limit its application *in vivo*.

#### MRS—^19^F

^31^P-MRS has 15 times less sensitivity in comparison to ^1^H spectroscopy; conversely, sensitivity of ^19^F is reasonably close to proton spectroscopy (83%) and its isotopic abundance is 100%. The main advantage of ^19^F-MRS for *in vivo* application relies on the minimal NMR background interference from endogenous signal and the large chemical shift range (~ 300 ppm) that allowed the development of several fluorinated probes able to report microenvironment changes of pO_2_, hypoxia, enzyme activity, and pH [24]. Aromatic molecules, such as the vitamin B6 analogue fluoropyridoxol, were reported for assessing pH *in vivo* thanks to the larger chemical shift response (~ 9.5 ppm) to changes in pH in comparison to fluoroalanine-based probes (~ 2 ppm) [25]. Early studies demonstrated the capability of 6-fluoropyridoxol (6-FPOL) to simultaneously measure the dynamic changes of pHe/pHi in perfused rat hearth with a time resolution of 2 min [24]. As the p*K*a (~ 8.2) of 6-FPOL is not ideal, a novel membrane-impermeant CF3-modified 6-trifluoromethylpyridoxine with a p*K*a in the physiological range was designed. This new molecule was successfully detected in mammary and prostate tumor xenografts allowing the measurement of the extracellular pH with a sensitivity of 0.40 ppm/pH unit [26]. However, fluorophenols have the drawback of ion-binding, thus limiting their application *in vivo*. An additional ^19^F-MRS probe, the fluoroaniline sulfonamide ZK-150471, has been validated as a valid pH reporter in different tumor xenograft models. The aromatic fluorine signal of ZK-150471 showed a chemical shift highly dependent on pH, whereas the trifluoromethyl group served as the intramolecular pH-independent reference. *In vivo*, ZK-150471 demonstrated to distribute only within the extracellular space of the perfused regions, showing good correspondence with microelectrodes pHe measurements [27]. Further, the capability of ZK-150471 to report pHe was compared with the ^31^P-MRS probe 3-APP in tumors. Although pHe values were not significantly different, the fluorinated probe has the advantage of a lower toxicity, thus allowing the administration of higher doses for investigating pH heterogeneity in tumors. The main limitations for *in vivo* applications are the relative instability of fluorinated probes and their nonspecific accumulation in tissues due to their hydrophobicity. To overcome these issues, new formulations based on the encapsulation of ^19^F compounds have been proposed. Promising results were obtained with PEGylated nanogels that showed variation in size in accordance with pH changes, in the range of 6.8–7.3, indicating that extracellular pH can be indirectly estimated from measuring the diameter of nanoprobes by ^19^F-MRS [28]. Similarly, an on/off strategy based on micelles encapsulating ^19^F containing species allowed the encoding of narrow pH transition (0.25 pH unit) through barcode map generated from specific ^19^F signatures [29]. Despite the absence of a background signal that improves the specificity of this approach, the moderate sensitivity still limits the spatial resolution achievable by ^19^F-MRS, even at higher magnetic fields.

#### MRS—^1^H

To overcome both ^31^P- and ^19^F-MRS issues, proton spectroscopy represents a valid alternative in terms of increased sensitivity due to its highest gyromagnetic ratio and a natural abundance of 99.98%. These properties represent a great advantage for gaining in spatial resolution with reduced acquisition time but a technical challenge since the broad and intense peak arising from the bulk water molecules need to be nullified for observing the smaller resonances of the exploited probes. For instance, a ^1^H-NMR pH-sensitive probe was reported by Aime and coworkers, exploiting a paramagnetic complex of Ytterbium, a lanthanide ion able to induce large paramagnetic shifts of nearby nuclei [30]. This compound was shown *in vitro* to represent an excellent NMR pH indicator since the chemical shift separation between a selected pair of resonances is strongly pH dependent and not affected by changes in concentration or in ionic strength. Moreover, the NMR resonances of the paramagnetic complex cover a much wider chemical shift region than those of diamagnetic systems, providing higher pH sensitivity since the NMR resonances are easily distinguishable from that of endogenous molecules. Further details of the *in vivo* exploitation of the large ^1^H-chemical shift of paramagnetic metal complexes as pH indicators can be found in the PARACEST paragraph.

**Fig. 1:**
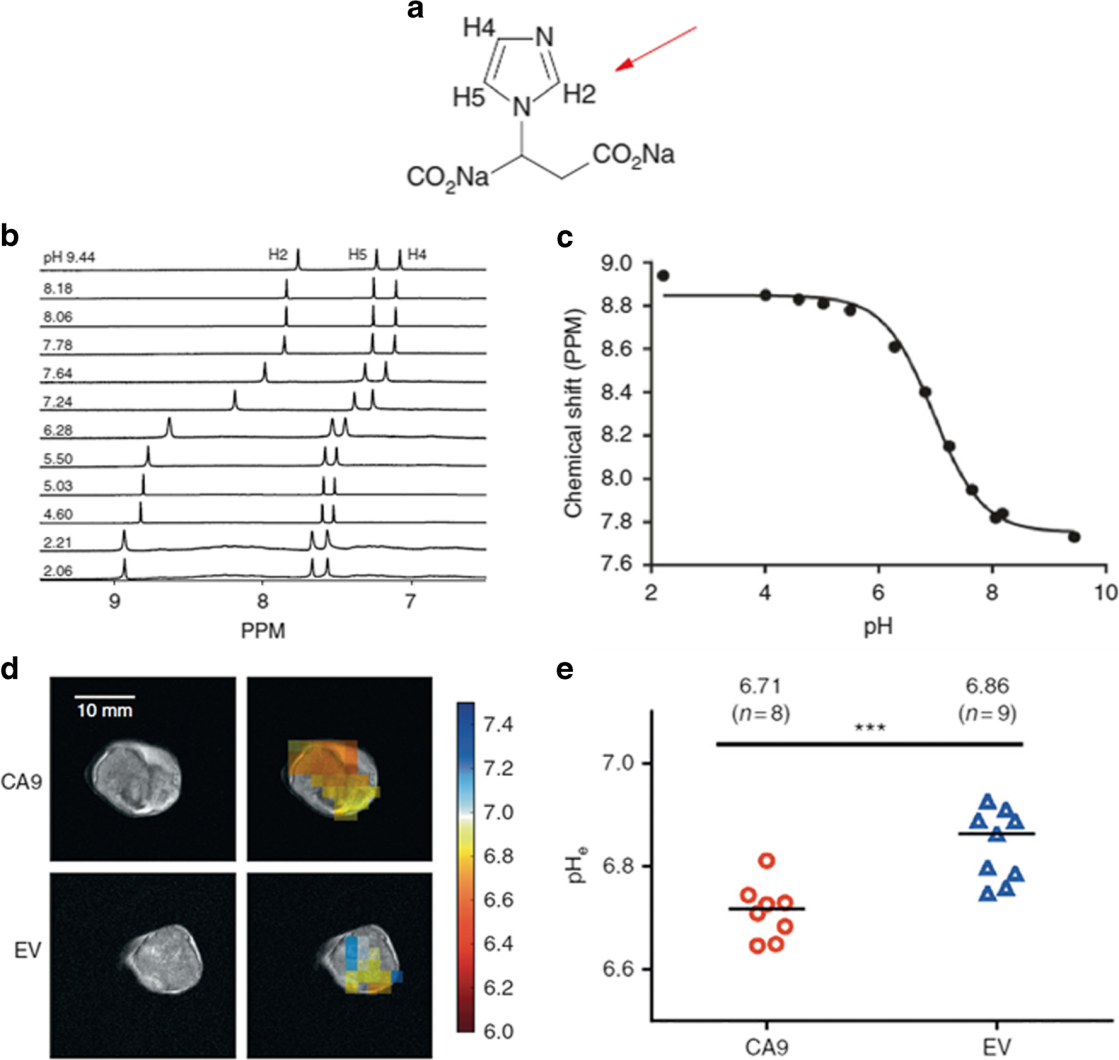
MRS imaging of tumor pH. Chemical formula of ISUCA and indications of proton resonances (pH-dependent H2, red arrow) on the imidazole ring (**a**). Plasmatic MR spectra of ISUCA at different pH values indicate the chemical shift dependency of H2 from pH changes (**b**). pH calibration curve of the chemical shift of H2 with changes in pH (**c**). Parametric pHe map over-imposed on *T*_2_-weighted MR images of high expressing CAIX (CA9) and low expression CAIX (EV) tumors (**d**) and median pHe value for CA9 and EV tumors (**e**). Adapted with permission from British Journal of Cancer 2018, 119, 622–630

For measuring *in vivo* pH by ^1^H-MRS, histidine and imidazole-containing molecules were initially considered due to (i) their p*K*a (~ 7.0) close to the physiological values and (ii) their proton NMR peaks that resonate enough away from the very intense water signal. First studies exploited histidine, a precursor of imidazole, since the presence of two resonances that show a pH-dependent chemical shift due to the protonation and deprotonation of the amine group [31]. Exploiting an oral histidine loading to increase the natural concentration of this molecule in the brain, they were able to report pH values in healthy human brains. The most significant results were achieved with 2-imidazole-1-yl-3-ethoxycarbonyl propionic acid (IEPA) that demonstrated to remain well confined in the extracellular environment. H2-IEPA shows a chemical shift that is highly dependent on pH with a titratable range of 7.8–8.9 ppm and a good pH accuracy of 0.1 pH units, thus providing the first *in vivo* tumor pHe map [32]. This approach was further explored in a C6 glioblastoma multiforme model implanted in rats with a spatial resolution of 1 × 1 × 4 mm^3^ [33]. In this study, IEPA demonstrated that a more acidic pH occurs in tumors in comparison to normal tissues. In addition, this approach allowed mapping the distribution of IEPA, metabolic compounds, and lactate within the same acquisition experiment. Although it was expected a spatial correlation with lactate and proton concentration, a local mismatch between the two measurements was reported. This result was further elaborated in a subsequent paper devoted to the use of a new imidazole-derivative, the (±)2-(imidazole-1-yl)succinic acid (ISUCA, Fig. 1a) that demonstrated superior pharmacokinetic properties in comparison to IEPA and a chemical shift strongly dependent on pH changes, Fig. 1b, c [34]. Similarly, a voxel by voxel comparison showed a different spatial distribution of acidity and lactate in gliomas, suggesting that intra- and extracellular flow of protons might influence acidosis at distant sites from lactate accumulation. More recently, ISUCA was exploited to monitor the activity of the isoenzyme carbonic anhydrase IX (see Pastorikova this volume) that produces H^+^ and HCO_3_^−^ in the extracellular environment from the hydration of CO_2_, and is associated with the most aggressive forms of cancer [35]. This mechanism of acidification has been validated *in vivo* on colorectal tumor xenografts with different expression levels of CAIX. Interestingly, mapping of pHe with ISUCA revealed that high CAIX expression decreased the steady state pH of the extracellular compartment in comparison to low CAIX expressing tumors, whereas no evident differences in pHi measurements by ^1^H-MRS were observed (Fig. 1d, e).

Although the accuracy and sensitivity on reporting pH changes offered by this method, the poor spatial resolution and the long acquisition times markedly affect pHe estimation and represent a limitation for accurate quantification of pHe heterogeneity. Another concern on using imidazole-based compound was raised due to the alkalization effect of non-volatile buffer that might affect pH measurements. Indeed, IEPA demonstrated buffering capabilities and chronic administration of IEPA influenced the extracellular acidity of tumors and reduced the formation of lung metastasis in an experimental model of prostate cancer [36]. In addition, the rapid elimination by renal clearance and the presence of only one H2 proton in IEPA and ISUCA limit the sensitivity of this approach for *in vivo* application. To overcome this issue, the design of diimidazole probes with higher polarity and double H2 resonance intensity is currently under investigation, with promising results for *in vivo* pHe measurements [37].

### MRI relaxometry-based pH-sensitive probes

Magnetic resonance imaging (MRI) has been widely used as a routine diagnostic tool in modern clinical medicine. The majority of clinically used MRI contrast agents are paramagnetic gadolinium (Gd^3+^) chelates, able to increase the signal intensity by shortening the longitudinal (*T*_1_) or the transversal (*T*_2_) relaxation times of water protons close to the region where the metal complex distributes [38–41].

**Fig. 2:**
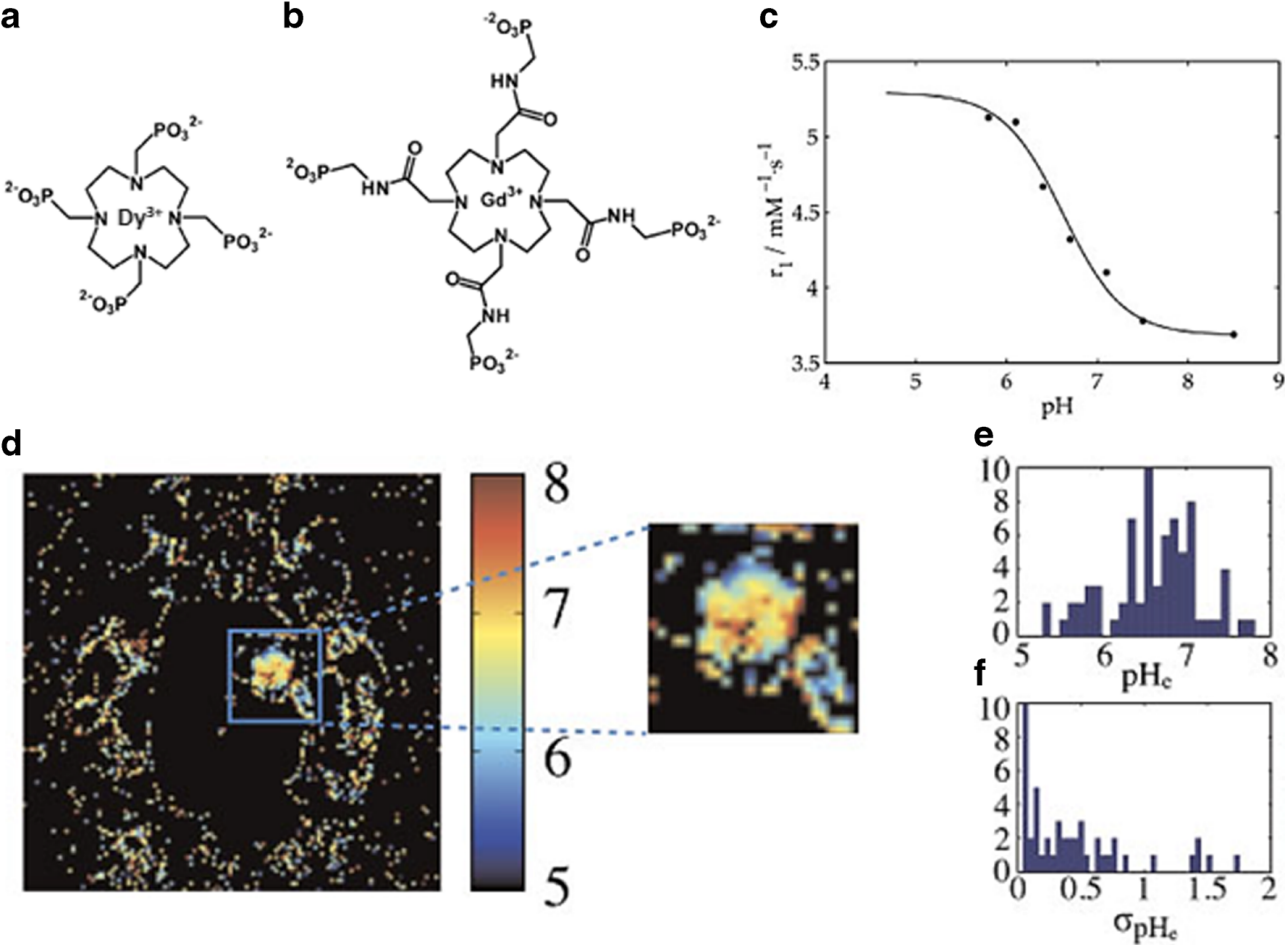
MRI-based relaxometry imaging of tumor pH. Chemical structure of the pH-insensitive *T*_2_ agent, Dy-DOTP (**a**) and of the pH-sensitive *T*_1_ agent, Gd-DOTA-4AmP (**b**). pH dependence of Gd-DOTA-4AmP relaxivity (**c**). Tumor pHe maps obtained following the injection of the cocktail of the two agents (**d**) and histograms of pH values (**e**) and standard deviations (**f**) within a region of interest. Reproduced with permission from NMR in Biomedicine 2011, 24, 1380–1391

pH-dependent paramagnetic metal complexes allow the evaluation of pH changes in extracellular tumor microenvironment by altering their *T*_1_ MR relaxation [42, 43]. The first approach to measure extracellular pH was described by Sherry and coworkers who exploited the protonation of the pendant arm of the Gd^3+^ complex of a tetraamide-based ligand. The changes in *T*_1_ relaxation times reflect changes in the number of inner sphere water molecules which is determined by the protonated/deprotonated state of the ligand [44]. The *in vivo* application of this pH-responsive agent was investigated with the tetraphosphonate gadolinium complex Gd-DOTA-4Amp in both C6 glioma and renal carcinoma [45, 46]. However, with this approach, changes in water proton relaxation times are dependent on both the concentration of the agent and its pH responsiveness; therefore, a direct readout of the solution pH is not possible. To overcome this limitation, a double injection of two similar contrast agents was proposed, one whose relaxivity is pH independent (Gd-DOTP) to estimate the concentration within the tissue of interest and the second one, with its relaxivity dependent on pH. By setting up a ratiometric method (i.e., by dividing the measured estimates), it is possible to calculate the pH. Unfortunately, the main drawbacks of this approach are as follows: (i) the administration of two contrast agents, (ii) one has to assume analogous pharmacokinetics and biodistribution of the two agents within the same anatomical region. A similar approach has been exploited following the simultaneous injection of two paramagnetic agents, in which the pH-sensitive Gd-DOTA-4Amp was mixed with the pH-insensitive *T*_2_^*^ dysprosium-DOTP agent (Fig. 2) [47]. Although this protocol allowed the investigators to obtain high-resolution pHe maps using relatively standard doses (0.2 mmol Gd/kg), still changes in *T*_2_^*^ need to be measured before and after the injection of the agents, assuming no competing effects in *T*_1_/*T*_2_^*^ times between the two contrast agents. Moreover, a similar biodistribution of the two probes within the tumor region must be proven for correctly quantifying tumor pHe. Other Gd-based agents have been investigated for mapping pH in a concentration-independent manner, but unfortunately without *in vivo* validation studies. A Gd-based complex covalently bound to an oligopeptide that undergoes pH-dependent configuration changes resulting in *T*_1_/*T*_2_ relaxation variations. Also, Aime and coworkers have demonstrated *in vitro* that Gd-DO3Asa-loaded liposomes provide a method for measuring pH by ratioing the measured relaxivities at different magnetic field strengths, opening new ways for relaxometric approaches [48, 49]. Other approaches, combining dual ^1^H and ^19^F containing probes using the heteronuclear ^19^F signal to normalize the contrast enhancement values due to a pH-sensitive Gd-complex, have also been investigated [50].

### MRI chemical exchange saturation transfer imaging

Chemical exchange saturation transfer (CEST) imaging is a MRI contrast mechanism that allows noninvasive detection of molecules or microenvironmental tumor properties such as pH, redox status, and enzymatic activity [51]. CEST method is based on the detection of changes of the water protons signal that decreases upon saturation magnetization at specific frequencies from an exchanging pool of protons, which can be from endogenous or exogenous sources. When RF pulses are applied, exchangeable protons from the contrast agent molecule become saturated and transfer their saturation to bulk water protons according to the chemical exchange rate, inducing a partial loss of net water signal. By applying a series of pulses, the saturation is transferred continuously to the water protons and is possible to detect the presence of molecules with mobile protons within MR images [52–54]. By recording the changes in the water signal intensity as a function of the applied RF pulses at different resonances (frequencies), one can obtain the so-called Z-spectrum that provides information on the exchanging molecule.

Proton exchange process depends on several factors, including concentration, temperature and, very often, pH. Many agents show an exchange rate that is usually slower at low pH than at high pH due to the occurrence of base-catalyzed proton exchange [55]. To date, several pH-responsive CEST MRI agents have been developed and in general, CEST has some advantages compared to other imaging modalities. First, CEST MRI has a great spatial resolution (less than 1 mm) that can provide tomographic images exploiting standard ^1^H coils available in clinical MRI scanners. In addition, in contrast to MRI-relaxivity methods, CEST can take advantage of the ratiometric approach for ruling out the concentration provided that in the same agent there are two different pools of exchanging protons. On the other hand, it suffers of limited sensitivity as it needs millimolar concentrations of mobile protons. The MRI-CEST approach can be further subdivided according to the chemistry of the molecule containing the exchanging protons in DIACEST (diamagnetic molecules, including both endogenous and exogenous molecules) or PARACEST (paramagnetic molecules, mainly represented by metal complexes) agents [56, 57]. The versatility of the CEST approach and probes enables different ways for measuring *in vivo* tumor acidosis.

#### Endogenous DIACEST pH methods

**Fig. 3:**
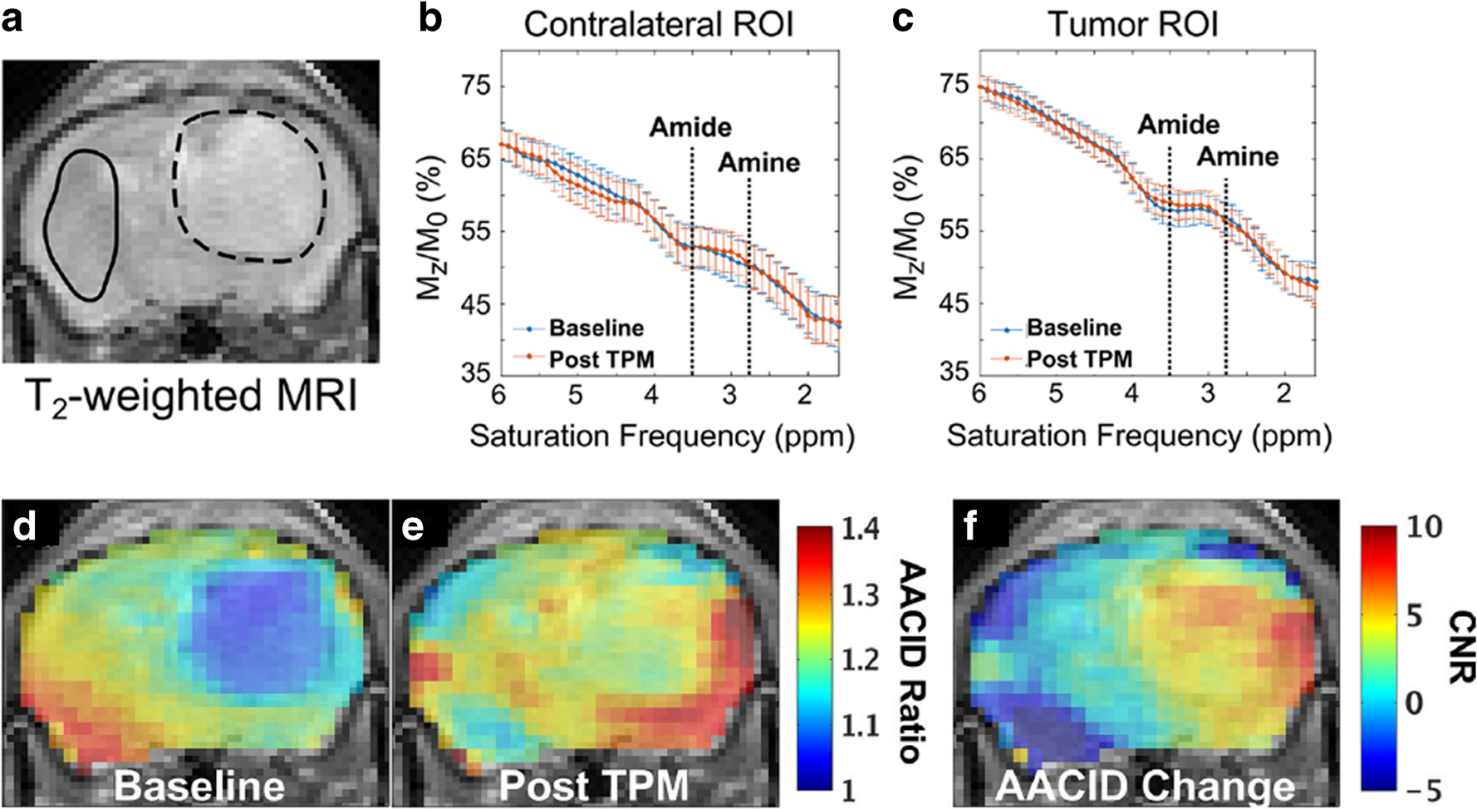
MRI-CEST imaging of tumor pH using endogenous DIACEST approach. *T*_2_-weighted MR image of a mouse brain with a U87 GBM tumor showing (**a)** region of interests (ROI) for the tumor region (dashed line) and for the contralateral region (solid line). CEST spectra from the contralateral (**b**) and from the tumor tissue (**c**) ROIs at baseline (blue) and ~ 75 min (red) after administration of 120 mg/kg of topiramate (TPM). The AACID measurement from the amine (2.75 ppm) and the amide (3.5 ppm) signal allows the calculation of the AACID maps before and ~ 75 min after i.p. injection of TPM (**d**, **e**, respectively) and the calculation of the AACID change map showing tumor selective acidification (**f**). Adapted with permission from Journal of Neuro-Oncology 2016, 130, 465–472

The chemical shift of typical endogenous diamagnetic exchangeable protons is within a few parts per million from bulk water protons (0–4 ppm) and since the exchange is base-catalyzed, the exchange rate is lower at acidic pH values. Amide protons contained in endogenous proteins and peptides have been the first source of mobile protons to be imaged and investigated for obtaining pH measurements according to their CEST contrast dependence from pH. This approach, called amide proton transfer (APT) imaging, has been exploited to obtain pH maps occurring in experimental models of brain stroke [58], since the reduced pH level in the ischemic region leads to a reduction in APT exchange rate, i.e., to a decrease of the CEST effect [59, 60]. This endogenous pH mapping method has also been shown to detect changes in pH following ischemia damage, but not yet applied to map tumor pH [61]. APT pH mapping has some limitations, since it is not possible to distinguish between the intracellular and extracellular contribution of amide mobile protons; hence, the derived pH values are a weighted estimate of both intracellular and extracellular pH. Secondly, the concentration of amide protons may vary in tumor; therefore, accurate pH values cannot be calculated. To overcome these limitations, Sun and colleagues have proposed and investigated different correction approaches to isolate the exchange rate (i.e., pH-weighted) contribution in the human brain [62–65]. On the other hand, Bartha’s group set up an amine/amide concentration-independent detection method (AACID) based on a ratiometric approach [66]. CEST effects originating at 2.75 ppm for amine groups and at 3.5 ppm for amide protons belonging to endogenous mobile peptides and proteins are ratioed (i.e., divided), allowing removal of the concentration term from the calculated pH values. With the AACID approach, they were able to detect *in vivo* intracellular pH changes after administration of different anticancer treatments in a glioblastoma murine model. Following the treatment with topiramate, an inhibitor of carbonic anhydrases, they observed a reduction of the intracellular tumor pH (corresponding to an increase in AACID values of 0.15 units) (Fig. 3) [67]. The method is sensitive enough to detect the slight changes in intracellular acidification of a implanted brain tumor following a single-dose administration of different drugs, such as lonidamine and dichloroacetate, targeting hexokinases and pyruvate dehydrogenase kinase, respectively [68, 69]. More recently, the same group applied this approach to monitor the therapeutic effect of quercetin, a monocarboxylate transporter inhibitor, on intracellular tumor acidification [70]. Since this approach does not require the administration of exogenous agents, it can be readily translated to the clinic. However, the abovementioned limitations of mixing intracellular and extracellular contribution on the calculated pH still hold true.

#### Exogenous DIACEST pH-responsive probes

Exogenous DIACEST agents have mobile protons with a chemical shift in the range 0–10 ppm from the water resonance. The main advantage in using exogenous agents relies on their capability to provide a net quantification of the extracellular tumor pH, since following the extravasation from the leaky tumor vasculature they remain in the extracellular extravascular space and do not enter the intracellular compartment. Iodinated or X-ray contrast agents, following the clever intuition of Silvio Aime [71], gained a lot of attention as DIACEST pH-sensitive agents for several reasons, namely, (i) the chemical structure of these molecules contains exchangeable amide protons that can be selectively saturated and provide CEST contrast in the 4–6 ppm range, far enough from water for a selective irradiation even at the magnetic field of 3 T; (ii) the CEST contrast is pH dependent and by applying a ratiometric approach, the pH measurement can be made concentration-independent; (iii) they are FDA-approved contrast agents used in the last 40 years for computed tomography (CT) investigations and have demonstrated a very high safety profile. Ongoing studies are also evaluating these molecules as alternatives to Gd-based agents for studying tumor perfusion [72, 73].

**Fig. 4:**
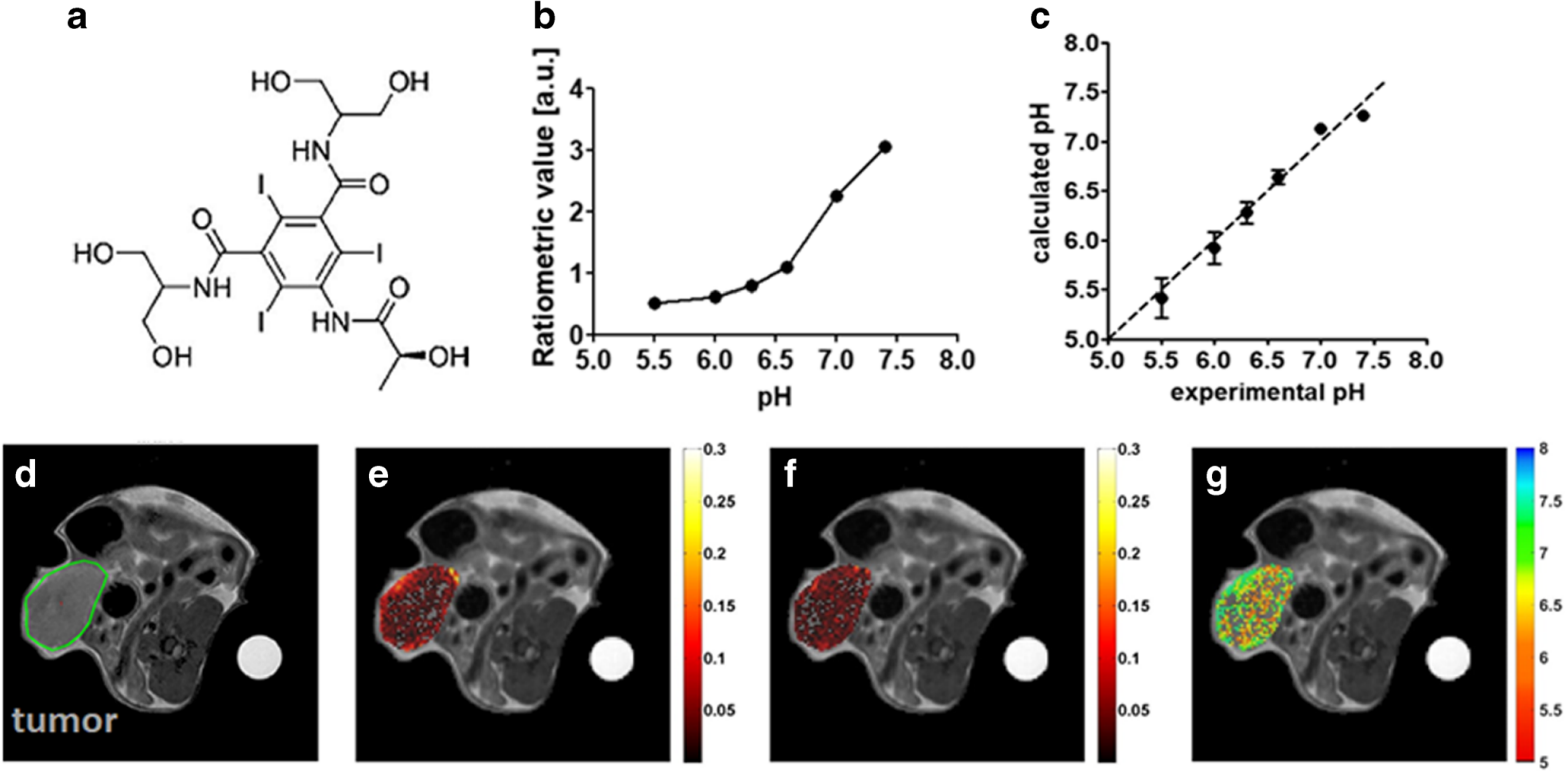
MRI-CEST imaging of tumor pH using exogenous DIACEST probes. Chemical structure of Iopamidol (**a**), pH dependence of the ratiometric values (**b**) calculated by ratioing the ST% contrast for the two amide groups (4.2 ppm and 5.5 ppm) and accuracy of the proposed method by comparing calculated vs. measured pH values (**c**) at 7 T. Adapted with permission by John Wiley and Sons from Magnetic Resonance in Medicine 2011, 65, 202–211. Representative *T*_2w_ anatomical images of a TS/A mammary bearing tumor mouse with tumor ROI highlighted in green (**d**). Contrast ST% difference map calculated at 4.2 ppm (**e**) and 5.5 ppm (**f**) after Iopamidol i.v. injection (as ST% post–ST% pre injection) and corresponding calculated tumor pHe map over-imposed on the anatomical image (**g**)

**Fig. 5:**
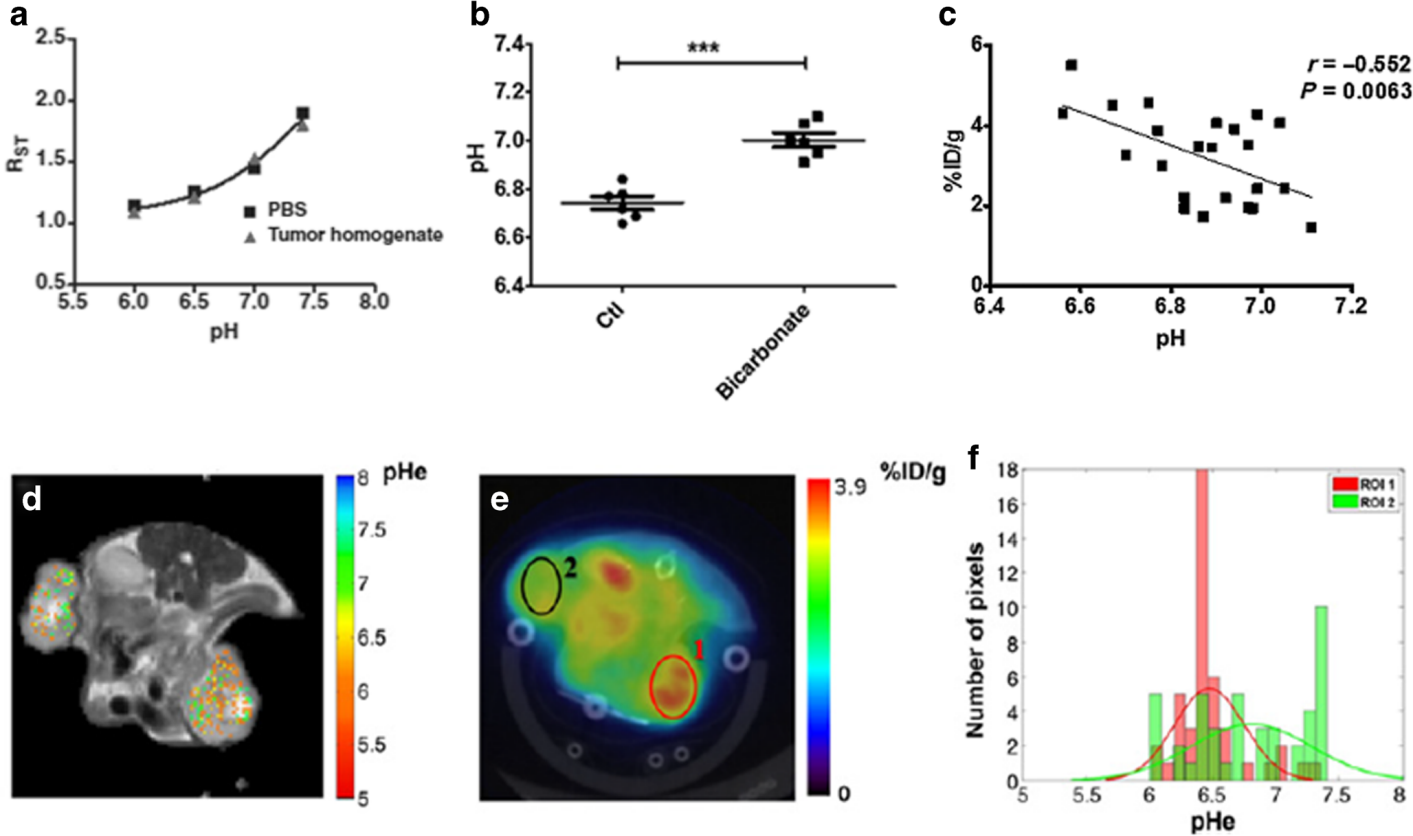
First *in vivo* demonstration of the relationship between increased glucose uptake and tumor acidosis. Plot of the pH dependence of the ratiometric (RST) values in the pH range from 6 to 7.4 for Iopamidol in phosphate buffer solution and in tumor tissue homogenate at 3 T (**a**). Average tumor pHe values calculated in mice drinking natural or sodium bicarbonated water showing a statistically significant increase of tumor pHe after 5 days (**b**). Scatterplots with regression line (solid line) showing a marked and significant correlation between ^18^F-FDG-PET %ID/g uptake and MRI-CEST-derived tumor acidosis (**c**). Combined MRI *T*_2w_/CEST pH (**d**) and ^18^F-FDG PET/CT (**e**) images of a representative TS/A tumor-bearing mouse. The tumor on the right side (ROI 1) shows a higher ^18^F-FDG uptake in the PET image, corresponding to more acidic pHe values in the MRI-CEST pHe map, whereas tumor on the left side (ROI 2) is characterized by lower ^18^F-FDG uptake values and corresponding less acidic pH values, as shown in the histogram plot (**g**). Adapted with permission from Cancer Research 2016, 76, 6463–6470

**Fig. 6:**
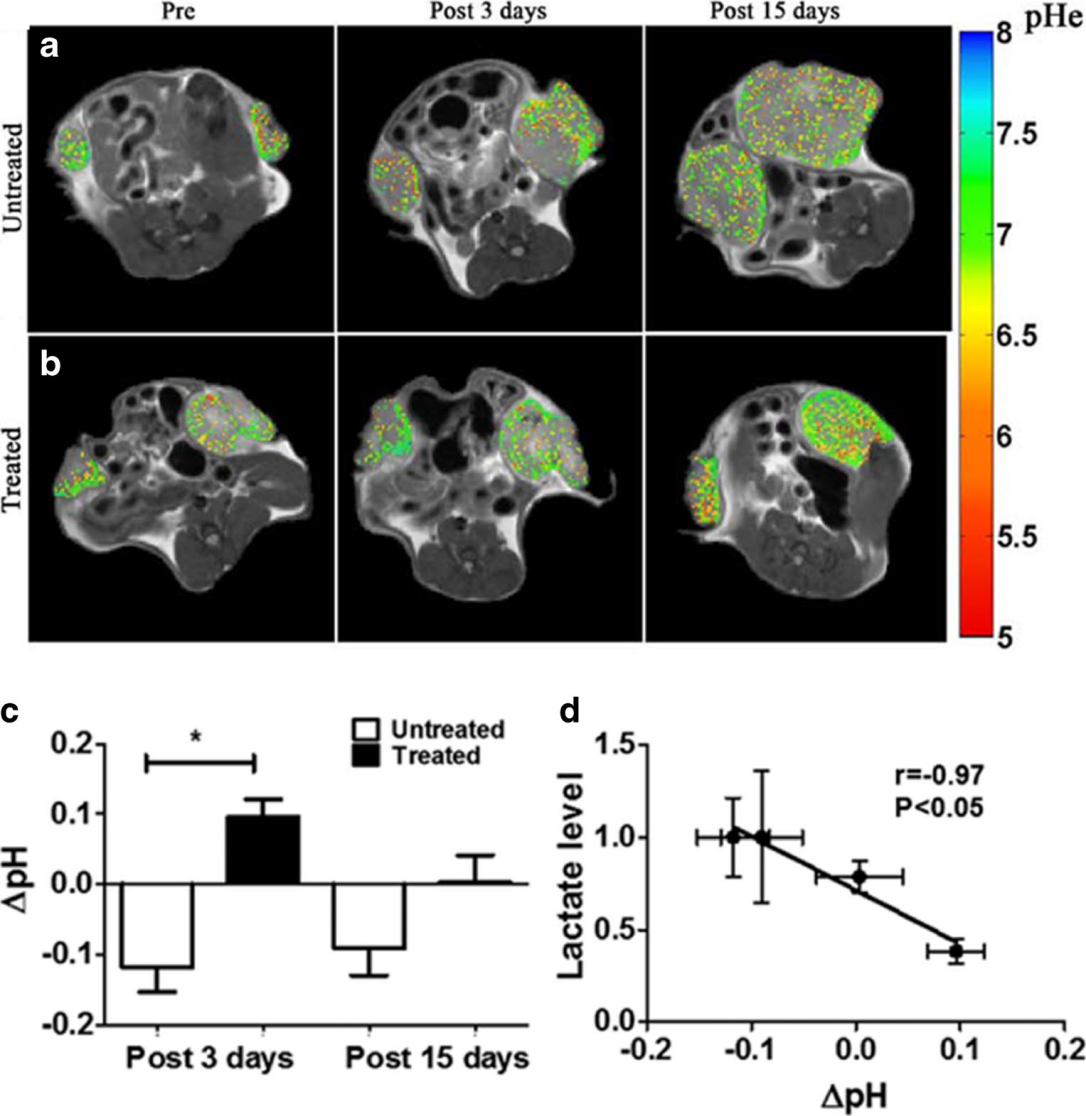
MRI-CEST tumor pH assessment of treatment response to novel anticancer therapies. Tumor extracellular pH maps (measured following Iopamidol injection) for representative mice at baseline, 3 days and 15 days for control group (**a**) or upon dichloroacetate (DCA) treatment (**b**) showing increased reduction of tumor acidosis. Changes in mean tumor pH values (in comparison to baseline tumor pH values) between untreated and treated mice with DCA at different time points (**c**) and correlation with lactate level measured in excised tumors (**d**). Adapted with permission from International Journal of Oncology 2017, 51, 498–506

Iopamidol (Isovue®, Bracco Diagnostics) was the first CT agent investigated as a MRI-CEST contrast agent, due to the high number of mobile protons (three amide protons and five hydroxyl protons) [74]. In particular, Iopamidol has two amide groups that resonate at 4.2 and 5.5 ppm, endowed with a pH-dependent exchange rate that allows to set up the proper ratiometric approach for measuring pH. In fact, it has been shown to provide good pH sensitivity and high pH accuracy in the 5.5–7.9 range (Fig. 4) [75, 76]. While the first *in vivo* validation dealt with measuring renal pH and the assessment of pH changes in several models of kidney injuries [77, 78], subsequent investigations demonstrated the usefulness of this approach in providing accurate tumor pHe maps. Notably, the first *in vivo* validation of the relationship between tumor dysregulated glycolysis and acidosis was reported by combining MRI-CEST tumor pHe mapping with Iopamidol and PET imaging following ^18^F-FDG injection [79]. This work showed the feasibility of pH mapping using a clinical magnetic field strength of 3 T and using homogenates of murine tumors for building the pH calibration curve (Fig. 5a). In addition, we showed that the method can monitor tumor pHe changes following sodium bicarbonate treatment (Fig. 5b), which neutralizes tumor acidity. Our group provided a clear evidence of the relationship between tumor metabolism and acidosis since tumor regions with higher FDG uptake displayed lower pHe values in a HER2 positive breast cancer model (Fig. 5c). Thanks to the high spatial resolution attainable by the MRI modality, the obtained tumor pHe maps showed the occurrence of a large heterogeneity in tumor acidosis that was not possible to investigate so far with other imaging modalities (Fig. 5d, e), therefore providing complementary information to the gold standard ^18^F-FDG PET for assessing tumor metabolism. Furthermore, it has been found that *in vivo* MRI-CEST pH mapping is a valuable tool for monitoring the therapeutic effect of novel anticancer therapies targeting tumor metabolism [10, 11, 80]. Dichloroacetate (DCA) is a small mitochondria-targeting molecule that is able to revert the glycolytic phenotype by blocking the pyruvate conversion to lactate, thus potentially reducing the extracellular tumor acidification [81]. Breast tumor-bearing mice treated with DCA showed a marked increase of tumor pHe values just 3 days after DCA administration, indicating the capability of CEST pH mapping to monitor early treatment effects that were associated with the decrease of lactate production (Fig. 6) [82]. Moreover, this approach was able to detect the onset of the resistance to the DCA treatment after 15 days, when tumor pHe returned to more acidic values. Overall, these results highlight the relationship between glycolysis, lactate, and acidosis and how the overall picture can be well investigated using MRI-CEST pH imaging.

**Fig. 7:**
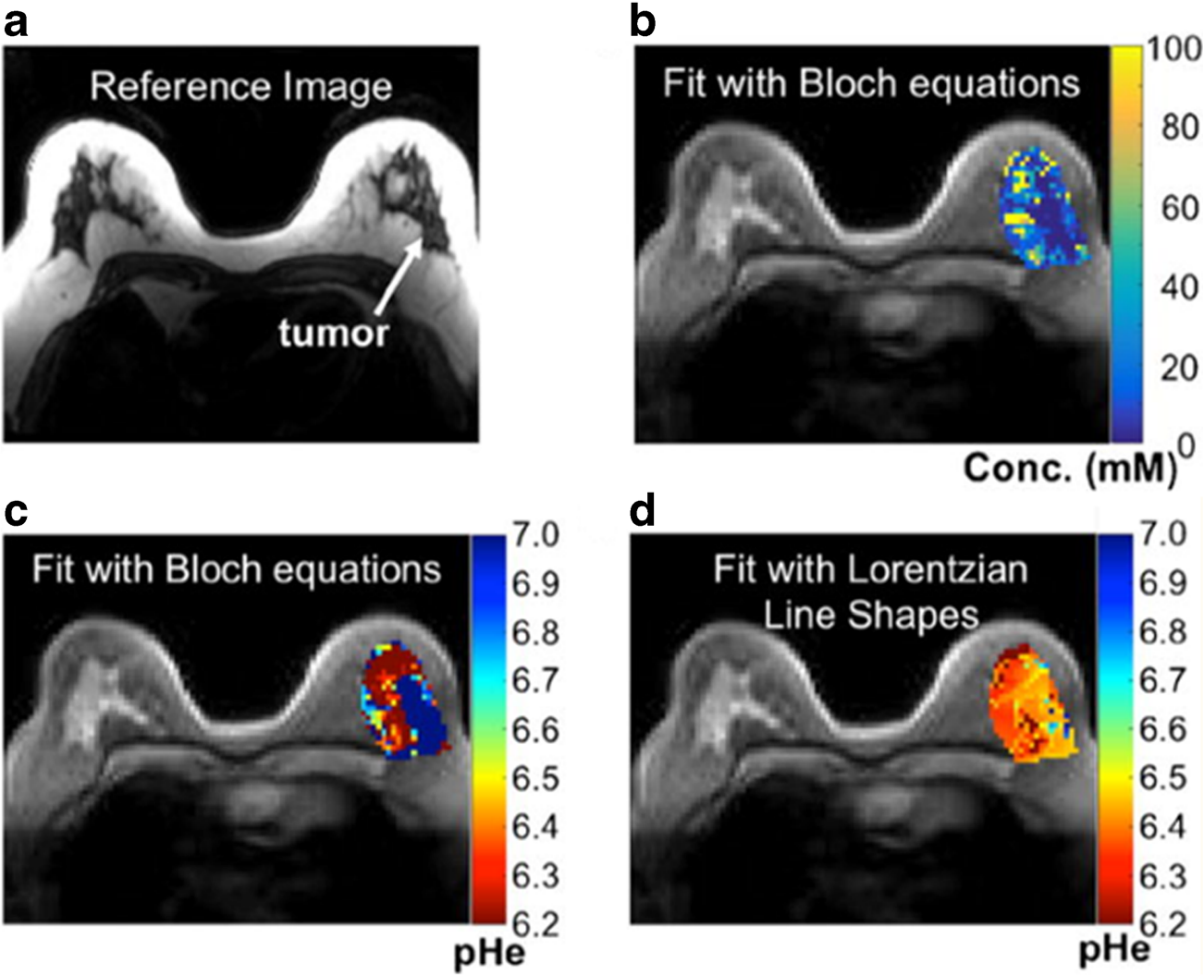
MRI-CEST imaging of tumor pH at clinical level. Representative patient with high-grade invasive ductal carcinoma and anatomical *T*_2_-weighted image (**a**) and parametric iopamidol concentration map (**b**) and tumor pHe map calculated by Bloch fitting (**c**) or by Lorentzian fitting (**d**) procedures overlaid on the anatomical image, showing, for the first time, tumor acidosis in patients. Adapted by permission from Springer Nature, Molecular Imaging and Biology 2017, 19, 617–625

An analogous validation of DIACEST tumor pH mapping has been accomplished by Pagel’s group, who used another pH-sensitive X-ray contrast agent, Iopromide (Ultravist®, Bayer Healthcare). Similarly to Iopamidol, the presence of two amide protons resonating at 4.2 and 5.6 ppm allows a ratiometric approach for measuring pH in the range 6.6 to 7.2 with good pH precision [83]. Additional studies demonstrated that tumor pHe maps were correlated with biomarkers associated with tumor metabolism, showing tumor pHe differences between preclinical models of mammary carcinoma [84]. *In vivo* comparative studies between Iopamidol and Iopromide in two different tumor murine models of Raji lymphoma and of MCF-7 breast cancer xenograft revealed that Iopamidol can detect more precisely the tumor pHe [85] and that Iopamidol pHe mapping combined with ^18^F-FDG PET can provide additional information for evaluating the therapeutic efficacy to metformin treatment in a pancreatic cancer model [86]. Since iodinated contrast media are approved for CT investigations, owing to the high safety profile, they have already been used in clinical trials for MRI-CEST pH mapping [87]. Of note, Iopamidol has been exploited for providing tumor pHe mapping in patients with ovarian or mammary cancers, showing, for the first time, clinical images of tumor acidosis (Fig. 7) [88]. Such important results are paving the way for further studies needed to confirm the diagnostic utility of tumor pHe mapping in the clinical scenario for both diagnostic and therapeutic monitoring.

**Fig. 8:**
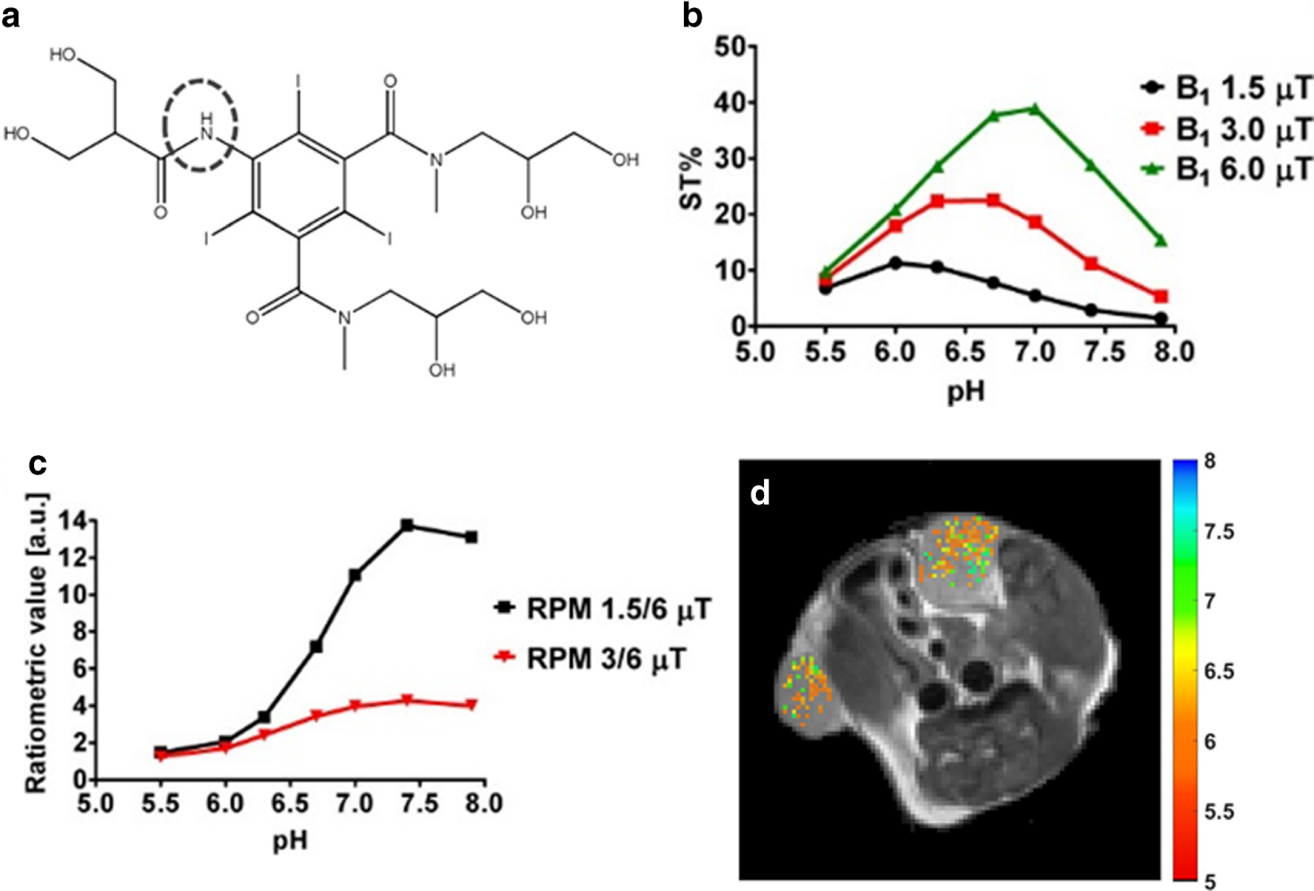
MRI-CEST imaging of tumor pH using exogenous DIACEST probes. Iobitridol chemical structure (**a**). Iobitridol MRI-CEST contrast (ST%) dependence with pH at three different RF saturation powers (**b**). Ratiometric approach between two different power levels allows to set up a pH calibration curve (**c**). Representative pH maps of a TS/A tumor-bearing mouse obtained upon rationing the difference ST map at 1.5 μT and at 6 μT after Iobitridol i.v. injection (**d**). Adapted with permission from Journal of the American Chemical Society 2014, 136, 14,333–14,336, https://pubs.acs.org/doi/10.1021/ja5059313. Copyright 2014 American Chemical Society

In the above-reported ratiometric approach, there is the requirement of two resonances with different chemical shift for carrying out their selective irradiation. To extend the capability of DIACEST molecules to act as pH sensors, a novel ratiometric approach for molecules possessing only one mobile proton pool (hence potentially transforming every CEST compound in a pH-sensitive agent) was developed. This approach has been validated by using another FDA-approved X-ray agent, Iobitridol (Xenetix®, Guerbet), with only one amide group resonating at 5.6 ppm (Fig. 8). It was shown that irradiating the same mobile proton pool with different power level, the measured contrast curves (with distinct pH dependence) can be ratioed, still preserving a good pH response in the pH range from 6 to 7.4 [89]. *In vivo* validation was demonstrated in a murine breast tumor model, where the measured pHe maps showed acidification of the tumor interstitial space, in good agreement with the corresponding Iopamidol-based tumor pHe maps. Further evolution of the power-based ratiometric approach has been demonstrated also for Iodixanol (Visipaque®, GE Healthcare), by exploiting a pulsed train saturation scheme [90].

Other pH sensors such as imidazole-4-5-dicarboxamides (I45DCs) have also been explored to provide CEST signal at frequencies quite far from water, up to 9 ppm, that can guarantee better detection. These systems have shown a good pH sensitivity and accuracy, but so far they have been exploited only for measuring pH in kidneys and not yet in tumors [91].

#### Paramagnetic pH-responsive probes (PARACEST)

**Fig. 9:**
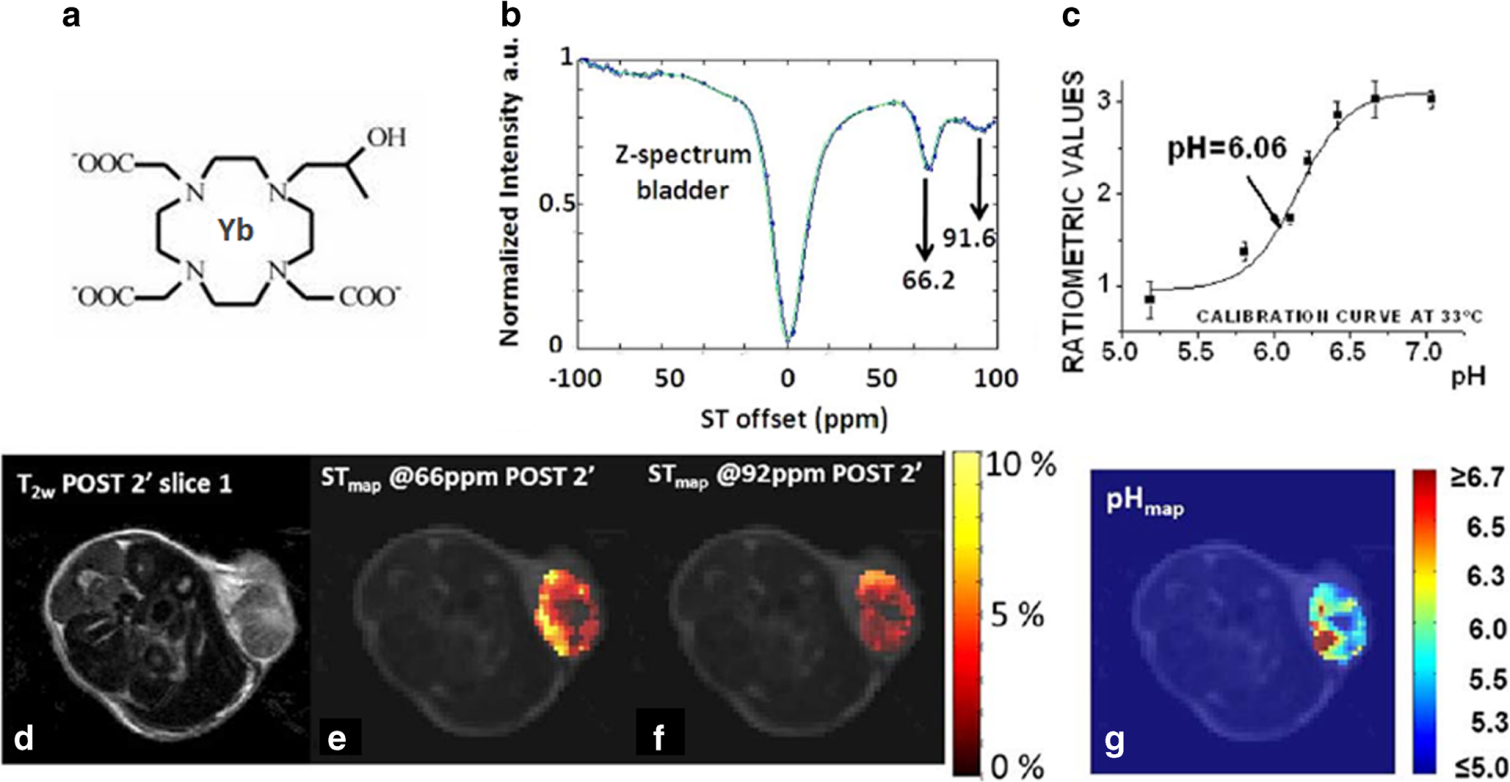
MRI-CEST imaging of tumor pH using exogenous PARACEST probes. Chemical structure of Yb-HPDO3A (**a**), Z-spectrum from the bladder region 15 min after the i.v. injection of Yb-HPDO3A (**b**) and pH calibration curve measured at 33 °C (**c**). *T*_2w_ image (**d**) and ST maps calculated when the irradiation offset is set at 66 ppm (**e**) and at 92 ppm (**f**) post 2 min after the i.v. injection of Yb-HPDO3A and measured tumor pH map (**g**). Adapted with permission from Magnetic Resonance in Medicine 2014, 71, 326–332

A significant limitation of DIACEST contrast agents is the small chemical shift separation between mobile protons and water resonances itself. This small frequency difference not only impedes detection, but it also limits the types of chemistries that can be used to keep the hydrogen exchange in the slow-to-intermediate time scale. In turn, this term is responsible for the reduced sensitivity of DIACEST agents, in particular at low magnetic fields. Paramagnetic CEST contrast agents (i.e., metal complexes of lanthanides or other metal cations) may display markedly increased chemical shift separations from the water resonance, which allow to access faster exchange rates before signal coalescence takes place [92]. The presence of mobile protons belonging to the coordinated water molecule or to chemical groups close to the coordinating metal cation has been exploited for generating the CEST contrast, which can be sensitized to a pH-dependent exchange rate. CEST properties of lanthanide (Ln = Eu, Dy, Ho, Er, Tm, Yb) complexes with the macrocyclic DOTAM-Gly ligand have been investigated for this purpose [93]. These complexes possess two groups of exchangeable protons at different chemical shifts: the pH-sensitive amide protons as well as the directly metal-coordinated water protons. Among the investigated Ln-complexes, Yb-DOTAM-Gly showed the highest pH responsiveness in the pH range 5.5–8.1. However, to provide pH measurement independent from concentration, a mixture of two complexes, Eu-DOTAM-Gly and Yb-DOTAM-Gly need to be used to set up a ratiometric method, although this approach has not yet been validated *in vivo*. A solution to overcome the problem of the double (or cocktail) injection (i.e., the occurrence of different pharmacokinetics and biodistribution of two agents) was presented by Delli Castelli et al. who proposed a ratiometric approach based on ratioing the saturation transfer effects from the amide group (pH sensitive) and water (pH independent) occurring in the same molecule [94]. Another solution was proposed with the Ytterbium (Yb) lanthanide analogue of the clinical Gd-based HPDO3A (Prohance) contrast agent that was considered as a PARACEST pH-responsive agent [95]. Within this molecule, the presence of two equilibrium diastereoisomers of Yb^3+^-HPDO3A in solution results in two CEST signals arising from the same hydroxyl proton close to the paramagnetic center with different MR absorption frequencies (77 and 91 ppm, respectively), thus allowing a ratiometric approach (Fig. 9). Yb^3+^-HPDO3A showed a pH sensitivity in the range 6.5–8.0 at a field strength of 7 T and demonstrated *in vivo* tumor pHe maps in a murine melanoma model [96]. In a recent study using Yb^3+^-HPDO3A, it was shown that tumor pHe mapping can also provide information about tumor aggressiveness in a rat glioma model [97]. The advantage of PARACEST agents endowed with highly shifted resonances is further witnessed in the case of Tm^3+^-HPDO3A pH-sensitive agent, which allows *in vivo* tumor pH mapping at lower magnetic fields (~ 1 T), due to the far-shifted hydroxyl resonances of the two diastereoisomers (71 and 185 ppm, respectively) [98]. PARACEST agents possessing two sets of exchangeable protons in the same structure were also investigated by Pagel’s group who reported that, in Yb^3+^DO3A-oAA, a complex containing both amide and aryl amine protons, the formation of intramolecular hydrogen bond with a proximal carboxylate ligand resulted in a reduction of the amine exchange rate, thus generating a second CEST effect [99]. By combining high magnetic field and moderate power saturation levels, intratumoral injection of the paramagnetic agent was used to produce a parametric map of tumor pHe.

Since PARACEST agents display resonances with large chemical shifts, the position of the resonating peak(s) can be exploited as an alternative approach for reporting on pH values. Sherry and coworkers developed a Europium-based contrast agent possessing a quite large shift in frequency of the metal-bound water molecule due to the delocalization of negative charge coming from the deprotonation of phenolic residue [100]. This process is driven by pH changes, where a change from 6.0 to 7.6 at 298 K leads to a shift for the water ^1^H-signal of 4 ppm, i.e., from 50.5 to 54.5 ppm respectively. By recording the CEST spectrum on a 9.4-T scanner, the measurement of the chemical shift of the absorbance peak provides direct estimates of tissue pH in a concentration-independent manner without the need to set up a ratiometric approach [101]. Although promising, it has not yet been validated for measuring tumor pHe. Moreover, since PARACEST agents are sensitive to temperature changes, accurate maintenance of temperature homogeneity is necessary for a correct pH estimation.

As the PARACEST method detects proton exchange between bulk water and any exchangeable sites (on the ligand itself or the inner sphere of bound water) that are shifted by the paramagnetic Ln^3+^ ion, another approach has been exploited interrogating non-exchangeable protons, in line with the early report of Aime et al. [30]. This method, dubbed biosensor imaging of redundant deviation in shifts (BIRDS), utilizes shifts of non-exchangeable protons from macrocyclic chelates complexed with paramagnetic lanthanide ions to generate pHe maps [102, 103]. The most recent application of the BIRD approach used TmDOTP^−^ to investigate tumor pHe differences in two glioma rat models, showing more acidic peritumoral pHe values in the more aggressive tumors [104]. A major concern regarding this approach was that renal ligation was needed to stop the renal clearance of the agent in order to allow a higher accumulation inside the tumor and, consequently, get higher sensitivity. In addition, very high magnetic fields (11.7 T) were needed to finely resolve small shifts in the resonances, limiting the attainable pH accuracy.

As a final remark on PARACEST agents, one may note that they are more sensitive than DIACEST agents as they are not influenced by the water-indirect saturation or by concomitant effects arising from the endogenous semisolid protein-based components. However, high doses are still required to reach sufficient concentrations at the region of interest for attaining detectable CEST signals, as well as high power levels are required for an efficient saturation of the fast-exchanging protons, which is not readily available on clinical scanners.

### MRI—spin-lock

**Fig. 10:**
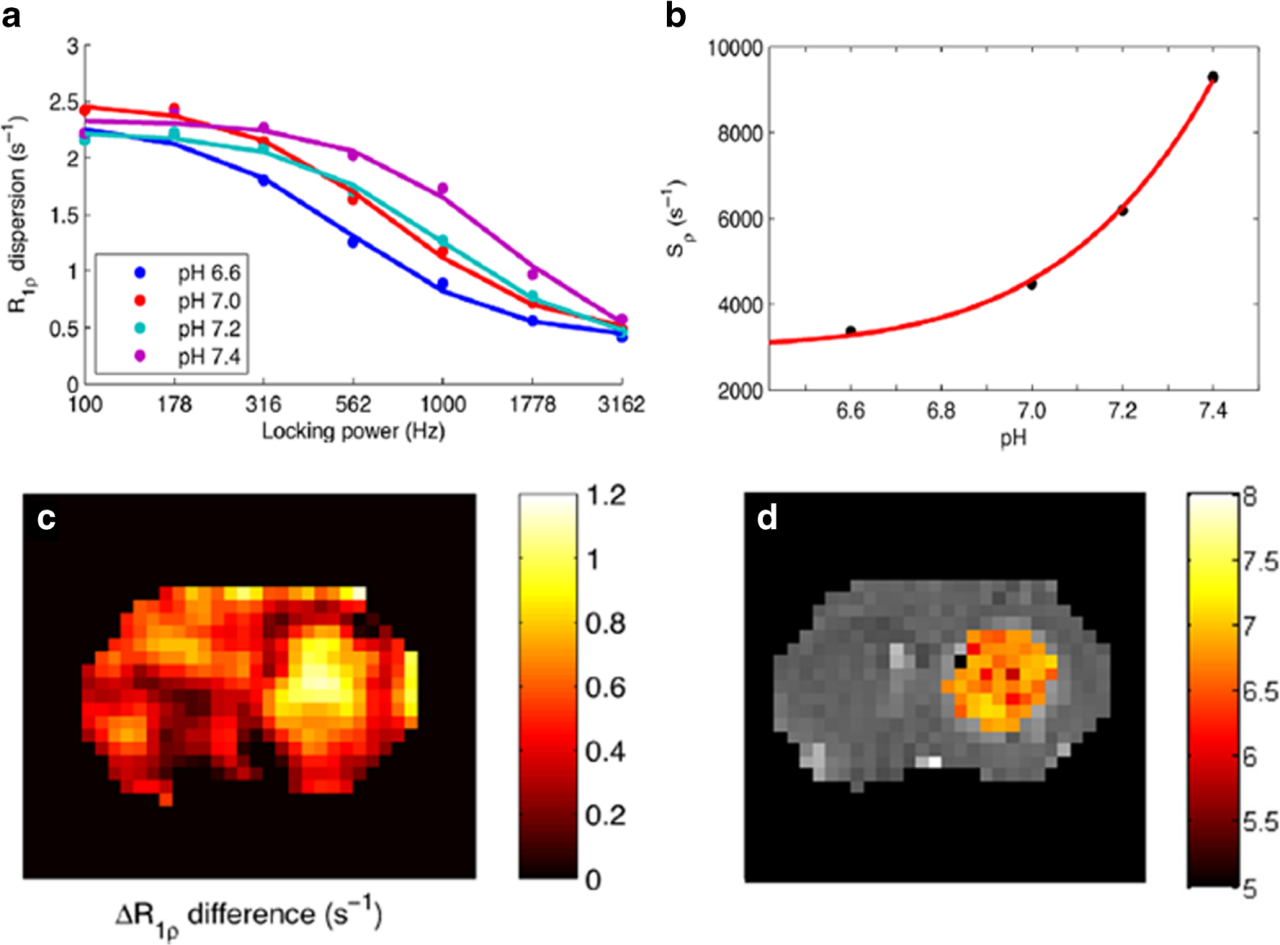
MRI-spin-lock imaging of tumor pH. Measured *R*_1*ρ*_ dispersion curves for Iohexol at several pH values but constant agent concentration (**a**) and calibration curve for the fitted s0 parameter as a function of pH (**b**). Δ*R*_1*ρ*_ difference map at 10 min after injection (**c**) and corresponding pH map from the tumor-bearing rat brain calculated as average of six Δ*R*_1*ρ*_ difference maps at different time points after iohexol i.v. injection (**d**). Adapted with permission from Magnetic Resonance in Medicine 2018, 79, 298–305

Besides the slow/intermediate exchange regime that is the basis of CEST, fast chemical exchange of mobile protons with water can be exploited for generating contrast in MR images, using a method based on the “spin-lock” (SL) technique [105]. With this approach, the water magnetization is first flipped from the *Z*-axis and then locked by radiofrequency pulses. During the applied SL pulse, the longitudinal water magnetization decay in the rotating frame (*T*_1*ρ*_) is sensitive to chemical exchange contributions, therefore providing an alternative method for exploring chemical exchange-based contrast [106]. In this context, molecules possessing fast-exchanging hydroxyl protons such as glucose can be detected more easily with the SL than with CEST [107, 108]. Iodinated X-ray agents possessing hydroxyl groups have also been investigated by the SL approach by Gore and coworkers [109]. The same group has exploited Iohexol (Omnipaque®, GE Healthcare), a CT contrast medium for mapping rat brain tumor pHe by exploiting the spin-lock approach at very high magnetic field (9.4 T) [110]. By measuring the differences in the *T*_1*ρ*_ dispersion profiles (i.e., a measure of *T*_1*ρ*_ values as a function of the power of the locking field) before and after the injection, and then fitting this Δ*T*_1*ρ*_ dispersion difference, it was possible to quantify the characteristics of the exchanging proton pools. According to a measured calibration curve, the tumor pHe values correspond to an extracellular pH ranging from 6.6 to 7.0 (Fig. 10). Compared with CEST imaging of X-ray contrast, the SL technique requires higher powers and longer acquisition times to improve image quality, but translation to clinical scanners may be feasible.

### MRI-hyperpolarized

Dynamic nuclear polarization (DNP) transfers polarization from electrons to nuclei, which provides a dramatic increase of sensitivity in MRS, particularly for low-γ nuclei, such as ^13^C [111]. A mix of ^13^C-labeled molecules with small quantities of a stable free radical cooled to ~ 1 K in a magnetic field and continuously irradiated at the electron paramagnetic resonance (EPR) absorption frequency of the radical species. This results in the transfer of polarization from the unpaired electron to the ^13^C nuclei [112]. Because the resonance frequencies of electrons are thousands of times higher than those of NMR nuclei, this transfer dramatically increases the polarization of the NMR nuclei, directly increasing the sensitivity theoretically up to 50,000-fold. In practice, increases in the signal-to-noise ratio of 10,000 are routinely achieved for DNP-hyperpolarized ^13^C-labeled molecules, allowing their direct detection with spectroscopic imaging, MRSI [113, 114]. The development of hyperpolarized molecules whose chemical shift is pH dependent can provide another method for *in vivo* pH imaging with high sensitivity.

Brindle et al. firstly described the injection of hyperpolarized ^13^C-bicarbonate as a pH-responsive agent, exploiting the acid-base equilibrium between H^13^CO_3_^−^ and ^13^CO_2_ that show distinct chemical shifts. According to the Henderson–Hasselbalch equation, the ratio of H^13^CO_3_^−^ and ^13^CO_2_ resonance intensities can be exploited for assessing pH. The method has been tested for mapping the extracellular pH in a lymphoma tumor murine model [115]. The pH map calculated after the intravenous injection of 100 mM hyperpolarized ^13^C-bicarbonate gave an average calculated pH of 6.71 ± 0.14. Scholl and coworkers applied this technique for studying temporal changes of tumor pHi and pHe during tumor growth in a rat glioma model [116]. Despite ^13^C-labeled bicarbonate has been initially proposed as a probe for tumor pHe imaging, the transport of CO_2_ and bicarbonate in and out the cells results in a mixed contribution of intra- and extracellular values to the measured pH.

**Fig. 11:**
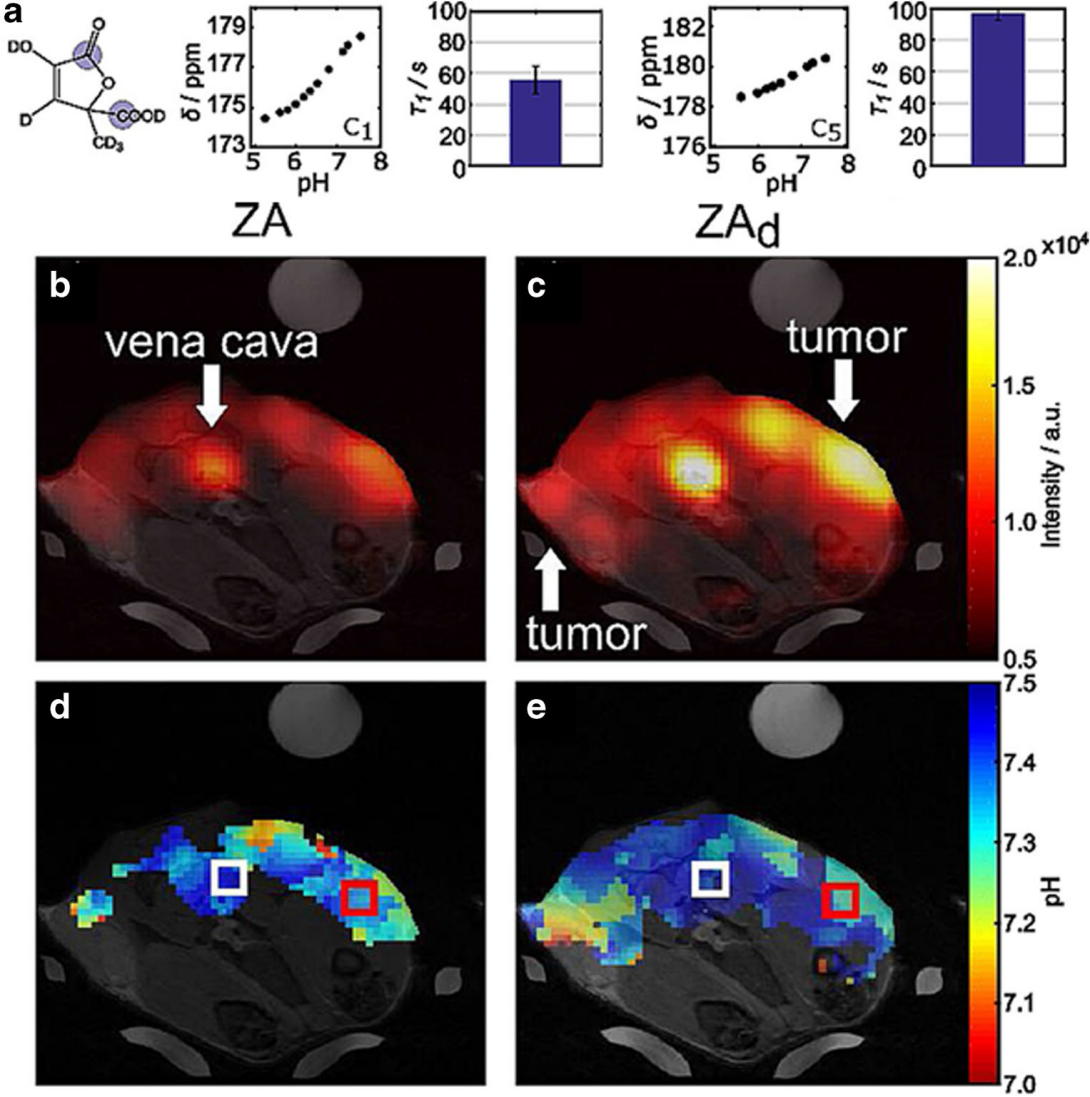
MRI-hyperpolarized imaging of tumor pH. Chemical structure of [1,5-^13^C_2_, 3,6,6,6-D_4_] zymonic acid (ZA_d_) with *T*_1_ values and pH-dependent chemical shifts of the C1 and C5 carbon atoms (**a**). *In vivo* signal intensity images acquired after tail-vein injection of ZA (**b**) and ZA_d_ (**c**) overlaid on anatomical *T*_1_-weighted ^1^H images. pH images calculated from the chemical shift differences of C5 and urea, weighted by the respective signal intensities, show an acidification of the tumor microenvironment for both ZA (**d**) and ZA_d_ (**e**). Reproduced with permission from ChemPhysChem 2017, 18, 2422–2425

Recently, a new pH-responsive probe has been introduced based on the chemical structure of the [1,5-^13^C_2_] zymonic acid (ZA) [117]. ZA was identified as an impurity within ^13^C-pyruvic acid and it was subsequently shown to act as a pH-sensitive molecule. The *in vivo* application described by Schwaiger, Schilling et al. of this new probe in mammary MAT B III tumors showed the detection of extravascular acid compartment by ZA signal that was in good agreement with three other independent pH measurements, suggesting the ability of ZA to measure and discriminate between extravascular pH and intravascular pH. The accuracy of pH measurement (0.1 pH unit) as well as the long signal lifetime (*T*_1_ of 17 s at 7 T *in vivo*) makes this new reporter valuable for further preclinical and clinical studies. Further, perdeuteration of ZA increased the T1 relaxation time significantly, prolonging hyperpolarization and increasing sensitivity [118], allowing dynamic monitoring of pH changes *in vivo* (Fig. 11). Likely, the development of novel MRI sequences for fast image acquisition and the improvement in signal detection by optimal design of the receiver coils will allow this technique to reach the sub-millimeter spatial resolution in the near future for high-resolution pH imaging. Unfortunately, the requirements of dedicated ^13^C coils and the technical challenges for producing, *via* DNP, hyperpolarized molecules still hamper the clinical adoption of this promising technique. Other routes for generating hyperpolarized molecules are currently under investigation as they may allow more versatile approaches to the field [119, 120].

## Electron paramagnetic resonance imaging

Electron paramagnetic resonance (EPR) is a spectroscopic technique based on the detection of unpaired electrons (radicals) in paramagnetic species, exposing the sample to a sweep of microwave frequency irradiation. Because of the insufficient amount of radical species in viable tissues combined with their fast relaxation, the use of paramagnetic probes to reach a sufficient concentration at the site of interest is required [121]. Whereas direct detection of paramagnetic probes guarantees high specificity, development of probes endowed with good biocompatibility, long stability during the measurement, low toxicity, and optimal spectral sensitivity is not a straightforward task. Moreover, unlike MRI measurements, EPR experiments are performed in a continuous wave mode, resulting in long acquisition times, small sample size, and restricted to surface tissues. EPR imaging (EPRI) is challenging for anatomic co-localization since, at low magnetic field, the small polarization of the ^1^H spin is not sufficient to generate MR images. Two approaches have been developed for imaging purposes, namely (i) the use of pulsed irradiations (pulsed-EPRI), which shortens the acquisition time [122], but still lacking of anatomical information, and (ii) the exploitation of the Overhauser effect. This technique, called OMRI (Overhauser enhanced magnetic resonance imaging), also known as proton electron double resonance imaging (PEDRI) was developed by Lurie et al. in 1988 [123]. In this case, the electron spin polarization is transferred to ^1^H polarization with subsequent MR image acquisition, resulting in an indirect detection of the radical probe [124]. Unlike EPRI, higher resolution can be achieved.

Khramtsov and coworkers have designed a pH-sensitive nitroxide R_1_ and its deutero-substituted analog, R_2_, for EPR monitoring of pHe in breast cancer models [125]. The enhanced stability of the proposed probe conjugated with its extracellular localization and pH sensitivity in the range of 6.5–7.5 allowed ready detection of pHe. The same group investigated a dual probe pTAM (deuterated derivative of the phosphonated triaryl methyl or TAM) for simultaneous pH and oxygen monitoring thanks to the extraordinary stability toward tissue redox processes, longer relaxation time, and narrower line width of this probe compared to nitroxide-based probes [126]. Suffering of the lack of EPR spectral information, the conventional PEDRI approach was recently improved with the variable field and variable radio frequency (VRF) PEDRI, where the pH map was obtained from two PEDRI acquisitions performed at the EPR frequencies of protonated and unprotonated form of a deuterated pH-sensitive probe, Im6, in Met-1 tumor-bearing mice [127]. The VRF PEDRI technique was applied to obtain the pH map distribution in tumor with an average value of extracellular pH of 6.8 ± 0.1 in agreement with the averaged pH values measured with the pH microelectrode.

**Fig. 12:**
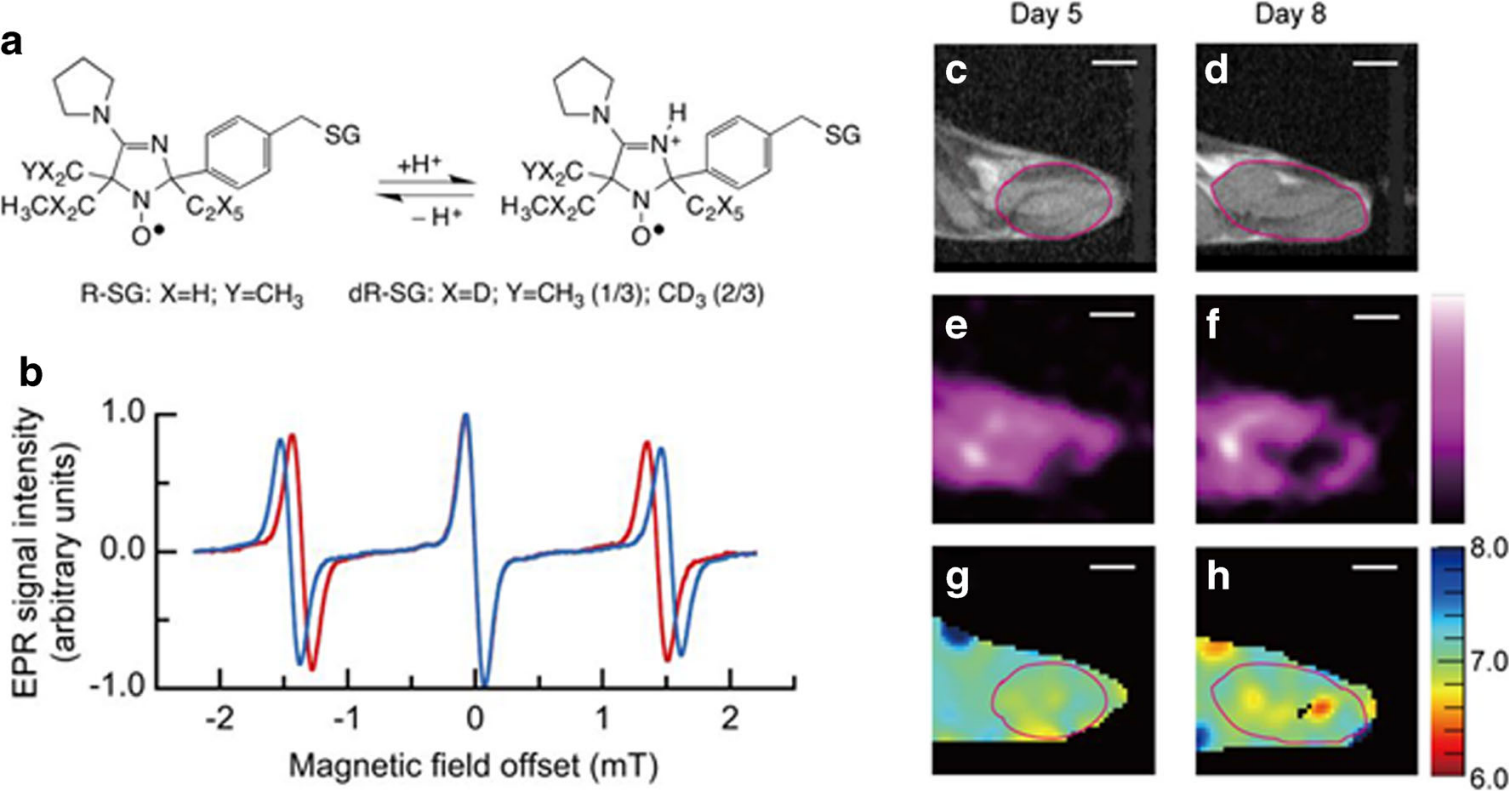
EPR-based imaging of tumor pH. Chemical structures and scheme of protonation of the pH-sensitive nitroxyl radical (R-SG) and its deuterium-enriched (dR-SG) analog (**a**). First derivative EPR spectra of 2 mM dR-SG measured at 750 MHz in alkaline (pH = 10.0, blue line) and acidic (pH = 3.0, red line) solutions (**b**). *T*_2_-weighted proton MR anatomical images of the SCC VII tumor-bearing mouse leg in the sagittal plane, acquired at day 5 (**c**) and 8 (**d**), respectively, and representative images of EPR signal intensity reconstructed from the 3D data (**e**, **f**) and corresponding tumor pHe maps at the two time points (**g**, **h**). The white scale bar on the images corresponds to 5 mm. Adapted with permission from Analytical Chemistry 2018, 90, 13,938–13,945

More recently, Komarov and coworkers implemented three-dimensional (3D) EPR pH mapping in animal models by applying fast projection scanning with a constant magnetic field. Both dedicated instrumentations and complex reconstruction algorithms are needed for deriving pH images from the measured EPR spectra, following the administration of a pH-sensitive nitroxyl radical probe (dR-SG) [128]. This newly proposed EPR method was demonstrated in mapping pH of tumor progression in a murine squamous cell carcinoma model (Fig. 12).

EPR approaches are promising in offering accurate pH measurements and multiple functional information, beside tumor pH, simultaneously. However, several drawbacks, including low spatial resolution (2–3 mm^3^ voxels, comparable to MRS), possible toxicity of the radical probes at the high required doses, demanding technical requirements, and high power radiofrequency irradiations, need to be solved before a large adoption of this approach can be foreseen.

## Positron emission tomography imaging

**Fig. 13:**
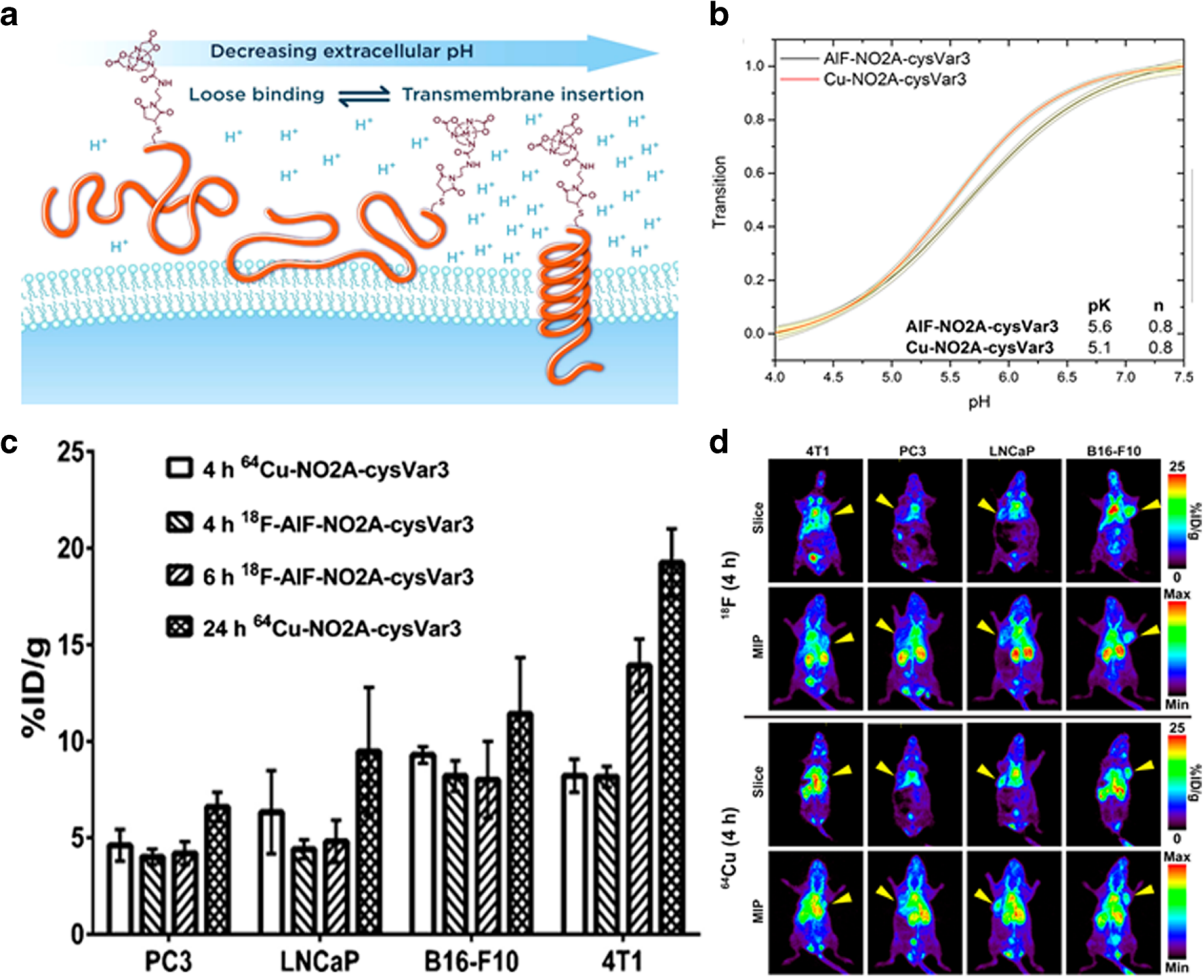
PET-based imaging of tumor pH. Schematic cartoon of the different folding states of pHLIP peptide in decreasing extracellular pH conditions (**a**). pH-dependent bilayer insertion of NO2A-cysVar3 constructs (**b**) and graph (**c**) of the *ex vivo* tumor uptake (%ID/g) for different radiolabeled peptides (^64^Cu and ^18^F) in several tumor models (prostate: PC3 and LNCaP, melanoma: B16F10, and breast: 4T1) and time points (4, 6, and 24 h). Slices and maximum intensity projections (MIP) PET images of radiolabeled peptides in tumor models at 4 h (**d**). The yellow arrowheads indicate where the tumor is located in each mouse. Adapted with permission from Bioconjugate Chemistry 2016, 27, 2014–2023

Positron emission tomography (PET) imaging is widely used in both preclinical and clinical imaging. It is characterized by a high sensitivity and limitless penetration depth and allows absolute quantification of the concentration of the injected tracer. However, PET is characterized by a low spatial resolution (~ 2 mm^3^) that limits its application for evaluation of heterogeneity in tumors [129]. Until now, only few pH-sensitive probes have been designed and tested, but have not yet been able to provide quantitative tumor pH images. Indeed, these probes can report on the presence of acidic extracellular environment only on the basis of their preferential accumulation, instead of providing a readout of the actual pH values. The first of these pH-responsive probe was a peptide (pHLIP) that changes its conformation in a pH-dependent manner (from random coil to alpha-helix), promoting its penetration into the cellular membranes at low extracellular pH values (Fig. 13) [130]. *In vitro* tests showed that at pH 7.4 less than 5% of the compound is incorporated into the membrane, while at pH 6, this value is more than 25%. In a preliminary study, ^64^Cu-DOTA-pHLIP was successfully imaged in prostate tumors (LNCaP and PC-3) showing a higher uptake and retention in the LNCaP tumors, whereas the uptake was reduced in mice that received 7 days of bicarbonated water [131]. In another study, pHLIP was bound to two positron emitting radionuclides (^64^Cu and ^18^F) and tested in breast (4T1), prostatic (LNCaP and PC3), and melanoma (B16-f10) tumor models [132]. ^64^Cu has a moderately short half-life and high spatial resolution that is an advantage for clinical application; however, its production is complicated and expensive. Conversely, ^18^F has longer half-life but it is routinely produced at numerous sites worldwide. In the slower growing tumors (PC3 and LNCaP), the tumor environment is less acidic, resulting in a lower accumulation; conversely, in more acidic and faster growing 4T1 and B16-F10 tumors, the accumulation was higher. Alternatively to pHLIP, Flavell et al. proposed an acid-labile precursor imaging tracer based on ^18^F-FDG with a glycosylamine linkage. In an acidic environment, the probe decomposes to release the ^18^F-FDG that accumulates in the cancerous cells. The probe was tested in a PC3 xenograft tumor model, showing a similar tumor uptake when compared to ^18^F-FDG, but capable of distinguishing changes in tumor acidosis following bicarbonate treatment [133]. This new class of PET tracer represents a promising strategy for addressing tumor acidosis, with the advantage of using the ^18^F-FDG derivatives at tracer doses. However, the accumulation of this tracer is still dependent on the presence of GLUT transporters, therefore reducing the exploitation in less glucose avid tumors, as for ^18^F-FDG. Overall, the PET-based approaches can only provide qualitative information of tumor acidosis with limited spatial resolution.

## Optical imaging

Optical imaging is characterized by high in-plane resolution and sensitivity, and excellent sensitivity and temporal resolution. However, it is limited in depth penetration and quantification, due to the well-known issues of absorption, reflection, and refraction processes when the emitted photons travel within biological tissues [134]. Importantly, in the NIR range (650–900 nm), photons travel through tissue deeper than photons in the visible range, and this wavelength range, called *in vivo* optical window, has been extensively exploited for addressing biological questions following the development of fluorescent-responsive probes [135].

SNARF-1 (semi-napthorhodafluorescein-1) is a fluorescent molecule that exhibits a pH-dependent emission spectral shift from 580 to 640 nm. The ratio of the measured fluorescent signals between these two wavelengths allows accurate pH measurements. Since the excitation and emission wavelengths are in the visible range, this probe can be applied in cell cultures or in superficial tumors like in rabbit ear chambers [136] or in intravital microscopy with the support of a window chamber [8]. To date, several studies using the window chamber tumor model have addressed important questions regarding the role of tumor acidosis in driving tumor invasion. In particular, by using this probe, Gillies and coworkers demonstrated that regions of highest tumor invasion correspond to areas of lowest pH [9]. On this basis, the administration of sodium bicarbonate to reduce tumor acidity (as demonstrated by the increase of the measured tumor pH values by this approach) was proposed as a novel therapeutic strategy to reduce tumor metastases [137]. Although the dorsal skinfold window chamber is commonly used to evaluate the microvasculature in various settings *in vivo*, it has to be stressed that a surgical operation is needed for implanting the window chamber and this operation could influence angiogenic processes. Moreover, pH measurements *via* SNARF-1 can be affected by difference in temperature between the calibration curve and the imaged tissue, as well as upon interaction of the fluorescent probe with extracellular proteins that can affect the pH dependence of the fluorescent spectra [138].

To measure *in vivo* tumor pH without the support of a window chamber, different probes with excitation and emission wavelength in the NIR region have been developed. Most of these approaches require two fluorescent probes, one pH sensitive and one pH insensitive, to set up a ratiometric approach for measuring pH values independently from the probes’ concentration. The conjugation of the two fluorescent dyes to a nanosized carrier guarantees the same concentration of the two molecules, solving the limitations associated with the assumption of the similar pharmacokinetic and biodistribution. However, the increase in the size of the resulting adducts, according to the final size and charge of the nanosystem, may limit the extravasation in poor perfused tumors or promote macrophage uptake and reduce the elimination rate, raising obvious toxicity issues. As an example, a pH-sensitive cyanine dye (CypHer5E), and a pH-insensitive fluorescent dye (Oyster800) have been conjugated on the surface of a biocompatible sphere with a diameter of 100 nm. CypHer5E has minimal fluorescence at neutral pH but becomes highly fluorescent with an emission peak at about 670 nm in an acidic environment. By taking the ratio of fluorescence intensities at different wavelengths, it was possible to detect tumor pH changes during tumor progression in a melanoma (B16f10) tumor murine model [139].

**Fig. 14:**
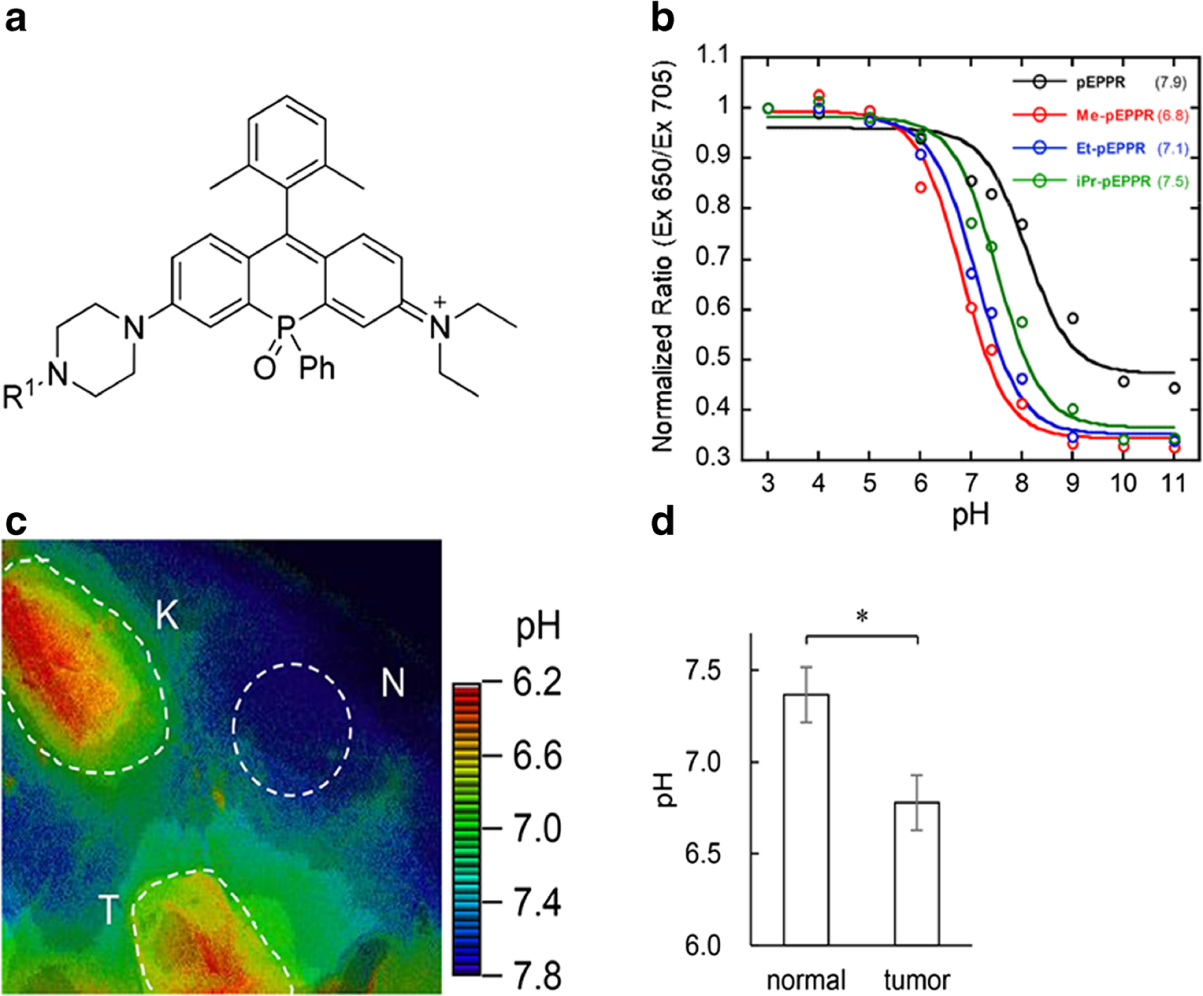
Optical imaging of tumor pH. Chemical structure of phosphorus-substituted rhodamine-based probe (**a**). pH calibration curves of several substituted rhodamine probes based on the ratio of fluorescence intensity at 650 nm and 705 nm with p*K*a values of the probes given in parentheses (**b**). *In vivo* fluorescence ratio images of mice with subcutaneous tumors (**c**, K kidney, N normal tissue, T tumor). Graph of mean pH values for normal tissue and tumor (**d**). Adapted with permission from Journal of the American Chemical Society 2018, 140, 5925–5933, doi:10.1021/jacs.8b00277. Copyright 2018 American Chemical Society

Another pH-ratiometric approach can be set up by using fluorescent dyes displaying two absorption peaks, one pH independent and the second pH dependent, respectively. The ratio of the fluorescent emission signals following the excitation at these two peaks makes the pH measurement independent of concentration. Besides the SNARF-1 pH sensor discussed above, rhodamine molecules were also proposed, with promising properties thanks to strong resistance to photobleaching, high water solubility, and high fluorescence quantum yields for both the protonated and deprotonated forms. Recently, a modified rhodamine bound to dextran (Dex-Me-pEPPR) has been synthetized and tested as a ratiometric NIR pH-sensitive probe. The intravenous administration of Dex-Me-pEPPR showed a more acidic value in the tumor region compared to healthy region (Fig. 14), in good agreement with the pH values measured using a pH electrode [140].

Finally, a different class of NIR pH-sensitive probes composed of systems that are activated by acidic pH has been proposed. For example, Gao and coworkers synthetized a 30-nm nanoparticle made of a pH-sensitive copolymer and loaded with the Cy5.5 dye. At blood pH values (7.4), the fluorescence is quenched, but at acidic pH values (6.9), the micelles dissociate, resulting in a marked increase of the fluorescence signal that allows detection of the acidic microenvironment of tumors [141]. A similar approach has been adopted to detect tumor acidosis by exploiting two NIR fluorophores (IR783) conjugated *via* a flexible and acid liable linkage. At neutral pH conditions, the fluorescence of this probe is quenched due to intramolecular dimeric aggregate. In the acidic conditions of the tumor microenvironment, the cleavage of pH liable linkage with the concomitant disruption of aggregates results in a remarkable fluorescence enhancement with a high tumor/background ratio. The ratio changed more dramatically upon time in a more metastatic hepatic tumor model (HCCLM3-GFP) in comparison to a less metastatic one (HepG2) [142].

## Photoacoustic imaging

**Fig. 15:**
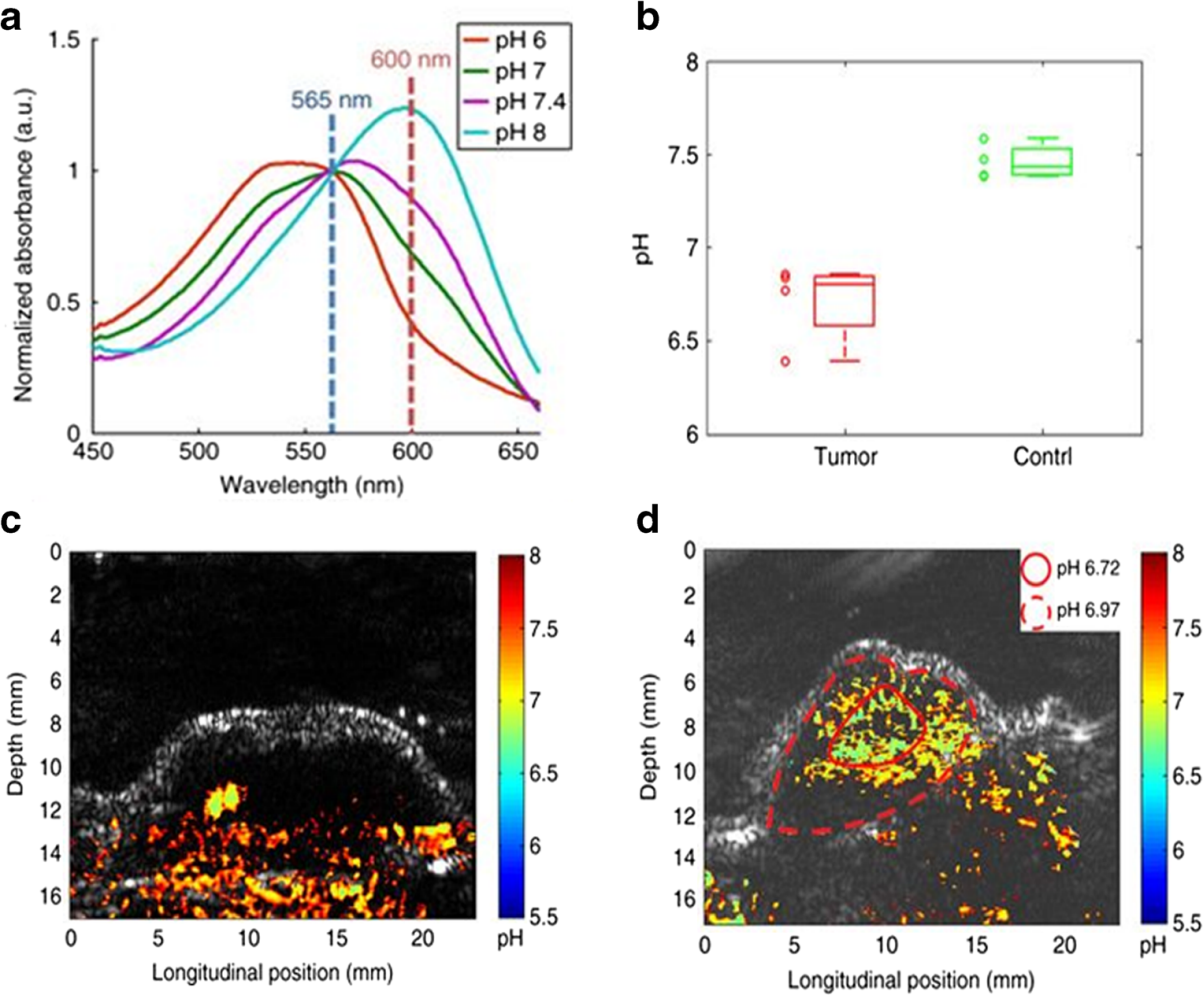
Photoacoustic imaging of tumor pH. Spectral properties of SNARF-5F encapsulated polyacrylamide based nanoparticle (NP) at different pH values (**a**). Boxplot showing the measured pH levels in tumor and healthy tissues (**b**) and quantitative PAI pH maps after SNARF-PAA NP injection in healthy (**c**) and in tumor (**d**) tissues overlaid to B-mode ultrasound images. Adapted with permission from Nature Communications 2017, 8, 471

Optoacoustic or photoacoustic imaging (PAI) combines optical absorption with ultrasound detection for providing high spatial and temporal resolution images [143, 144]. Since highly penetrant soundwaves are detected instead of light, optoacoustic imaging has greater penetration depth than optical imaging modalities [145]. Moreover, optoacoustic imaging offers greater specificity than conventional ultrasound due to detection of ultrasound emissions from light-absorbing chromophore-containing molecules, which can be exogenous probes, as well as endogenous systems such as hemoglobin, melanin, and lipids [146–149]. The ideal optoacoustic contrast agent should be characterized by a strong absorption in the optical window to maximize penetration depth and by a low quantum yield (the ratio of the number of photons emitted to the number of photons absorbed), as the non-radiative conversion of light energy to heat is maximized [150]. The capability of providing pH information is dependent on the absorption/emission properties of the fluorescent dyes; therefore, ratiometric approaches for measuring tumor pH such as those described above have been developed also for PAI probes. This task has been accomplished by assembling a pH-sensitive dye with a pH-inert dye in a nanoprobe. Benzo-α-phenoxazine (a pH-responsive dye) and IR825 (a pH-inert dye) can self-assemble onto human serum albumin (HSA) to form nanoparticles that allow assessment of tumor pH by ratioing signal intensities produced by excitations at 680 and 825 nm, for pH-sensitive and pH-insensitive emissions, respectively [151]. In a similar way, the pH-responsive near-infrared croconine dye can induce self-assembly on serum albumin nanoparticles for real-time ratiometric (680 and 810 nm) optoacoustic tumor pH imaging [152]. Other classes of nanosystems can be exploited by this approach, such as semiconducting oligomers that, following the binding with the pH-responsive boron-dipyrromethene dye, which can provide tumor pH mapping by ratioing the measured optoacoustic signals at 680 and 750 nm. It has been shown that this method readily distinguishes healthy and tumor tissues [153]. Polyacrylamide nanoparticles encapsulated with an optical pH indicator (SNARF-5F) have also been reported as pH-sensing nanoprobes. The capability of fast multi-wavelength PAI combined with spectral unmixing techniques can provide accurate tumor pH measurements not susceptible to the background optical absorption of endogenous biomolecules, i.e., oxy- and deoxy-hemoglobin (Fig. 15) [154].

The major limitations of these probes are related to the stability of the self-assembled nanoparticles that usually require further conjugation *via* glutaraldehyde to induce covalent cross-linking and to the toxicity profiles and biodistribution properties that need to be further optimized for their clinical translation. In addition, the nonlinear response of the ratiometric curve to changes in pH, coupled to the nonlinear optical properties of the photoacoustic effect, makes them more useful for detecting rapid or relative pH changes, rather than pH values. Despite these limitations, optoacoustic pH imaging overcomes the penetration depth limit of optical imaging, so it has the potential to measure tumor pH *in vivo* in superficial tumors up to 5–7-mm depth or in endoscopically accessible tumors at clinical level.

## Summary

A wide range of imaging-based techniques have been investigated to monitor tumor pH. In the last decade, several approaches have solved the limitations associated with first studies, including spatial and temporal resolution, pH sensitivity, and clinical translatability. To date, among the proposed techniques, MRI and in particular MRI-CEST methods have emerged as those endowed with good pH sensitivity for assessing tumor acidosis and pH changes following therapeutic treatments and with high spatial resolution for evaluating heterogeneity of the extracellular acidification. In addition, MRI-CEST using Iopamidol has been used to map tumor pH at clinical level, thus providing a novel imaging protocol for assessing fundamental questions in tumor biology, for evaluating the relationship between tumor acidosis and aggressiveness, and for monitoring treatment response to novel anticancer therapies. However, critical evaluation of this approach in single-site and multi-site studies should be encouraged to validate the clinical utility of imaging tumor acidosis as a novel noninvasive diagnostic tool.
